# Formulation, High Throughput *In Vitro* Screening and *In Vivo* Functional Characterization of Nanoemulsion-Based Intranasal Vaccine Adjuvants

**DOI:** 10.1371/journal.pone.0126120

**Published:** 2015-05-11

**Authors:** Pamela T. Wong, Pascale R. Leroueil, Douglas M. Smith, Susan Ciotti, Anna U. Bielinska, Katarzyna W. Janczak, Catherine H. Mullen, Jeffrey V. Groom, Erin M. Taylor, Crystal Passmore, Paul E. Makidon, Jessica J. O’Konek, Andrzej Myc, Tarek Hamouda, James R. Baker

**Affiliations:** 1 Michigan Nanotechnology Institute for Medicine and Biological Sciences and Department of Internal Medicine, University of Michigan Medical School, Ann Arbor, Michigan, United States of America; 2 NanoBio Corporation, Ann Arbor, Michigan, United States of America; The Ohio State University, UNITED STATES

## Abstract

Vaccine adjuvants have been reported to induce both mucosal and systemic immunity when applied to mucosal surfaces and this dual response appears important for protection against certain pathogens. Despite the potential advantages, however, no mucosal adjuvants are currently approved for human use. Evaluating compounds as mucosal adjuvants is a slow and costly process due to the need for lengthy animal immunogenicity studies. We have constructed a library of 112 intranasal adjuvant candidate formulations consisting of oil-in-water nanoemulsions that contain various cationic and nonionic surfactants. To facilitate adjuvant development we first evaluated this library in a series of high-throughput, *in vitro* assays for activities associated with innate and adaptive immune activation *in vivo*. These *in vitro* assays screened for the ability of the adjuvant to bind to mucin, induce cytotoxicity, facilitate antigen uptake in epithelial and dendritic cells, and activate cellular pathways. We then sought to determine how these parameters related to adjuvant activity *in vivo*. While the *in vitro* assays alone were not enough to predict the *in vivo* adjuvant activity completely, several interesting relationships were found with immune responses in mice. Furthermore, by varying the physicochemical properties of the surfactant components (charge, surfactant polar head size and hydrophobicity) and the surfactant blend ratio of the formulations, the strength and type of the immune response generated (T_H_1, T_H_2, T_H_17) could be modulated. These findings suggest the possibility of using high-throughput screens to aid in the design of custom adjuvants with unique immunological profiles to match specific mucosal vaccine applications.

## Introduction

Vaccines are a fundamental and important means of protection against pathogens. The mucosal route of vaccination can offer a number of advantages over intramuscular (IM) and subcutaneous administration, such as the potential to induce broader immune responses (including cellular, mucosal and systemic immunity) as opposed to the antibody responses that predominate with most parenteral vaccines [1–4]. For this reason, there is now an evolving consensus that mucosal vaccines will be necessary for effective prophylaxis against pathogens whose path of entry is through mucosal surfaces. As mucosal immune surfaces exhibit significant crosstalk, induction of an immune response at one mucosal site has been shown to elicit protective immunity at distant mucosal surfaces as well, making mucosal vaccines promising candidates for prophylaxis against sexually transmitted diseases [1, 5, 6]. Furthermore, these surfaces, such as the nasal mucosa, present a high density of immune cells which are easily accessible [6]. These potential benefits have spurred new interest in the development of mucosal vaccines; however, the number of new mucosal vaccines is limited in part because the mechanisms underlying mucosal immunity are not well understood. In addition, the lack of *in vitro* assays that can predict *in vivo* immunogenicity makes mucosal vaccine evaluation burdensome, as it necessitates lengthy immunization studies with large numbers of animals.

Nanoemulsions (NEs) are nanometer scale (d = 200–700 nm) oil-in-water emulsions composed of a combination of surfactants, a co-solvent (ethanol), oil (soybean oil), and water [7–9]. Our previous studies with a NE formulation containing the cationic surfactant cetylpyridinium chloride (CPC) and the nonionic surfactant, Tween80 have demonstrated that it can serve as an effective intranasal adjuvant for a variety of antigens and induce systemic antibody titers comparable to injected aluminum-based vaccines [8]. In contrast to injected aluminum-based IM vaccines however, this NE also induces systemic and mucosal T_H_1 and T_H_17 immunity [7, 10]. We have further demonstrated NE induction of protective immunity against a broad range of pathogens including hepatitis B, *Bacillus anthracis*, influenza and respiratory syncytial viruses, as well as adjuvanticity for HIV gp120 [7–9, 11–17]. The adjuvant activity of the CPC/Tween80 NE appears to require the presence of specific formulation components, as emulsions made with different surfactants demonstrated different patterns of immune activation *in vivo* or no adjuvant activity at all [18, 19].

While the NE mucosal adjuvant activity was initially discovered stochastically, the mechanism of this action is now being elucidated. This compound uniquely activates local cytokine production when applied to the nasal mucosa in mice, activating and trafficking dendritic cells to regional lymphoid tissue [18]. Furthermore, the NE also induces local apoptosis in ciliated epithelial cells *in vivo* and was shown to enhance antigen uptake in the nasal epithelium through increases in epithelial cell antigen uptake and through the phagocytosis of dead or dying antigen-loaded epithelial cells by dendritic cells [20]. This uniquely induces an interaction between innate activities in the mucosa and adaptive components of systemic immunity, potentially mimicking the immune activation activities of mucosal viruses.

In order to optimize the adjuvant activities of the NE and further understand the mechanism underlying NE induced immune activation, 112 NEs were produced containing combinations of 6 different quaternary amine cationic (or zwitterionic) surfactants with 18 different nonionic surfactants at various blend ratios. The surfactants were chosen such that they spanned a diverse range of physicochemical properties, including charge, hydrophobic tail length and polar head group size. We characterized the physical properties of the NEs and screened the stable formulations using *in vitro* assays for activities that appear important to adjuvant activity *in vivo*. These activities include stimulation of inflammation through cytotoxicity and immunogenic cell death [21, 22], facilitation of antigen uptake either through a depot effect (i.e. enhanced adherence to mucous) [19, 23–25] or through facilitation of antigen engulfment and presentation through phagocytosis [20, 26, 27], and activation of immune receptor mediated signaling such as the Toll-like receptor pathways [2, 3]. A remarkable range of activities were observed among the formulations *in vitro*, and select NEs were then evaluated *in vivo*. While some surfactant combinations generated robust immune responses in the form of antigen-specific antibodies, others did not, and the cellular response types (T_H_1, T_H_2, T_H_17) were highly dependent on the surfactant composition. It is clear that the factors influencing adjuvant activity *in vivo* are highly complex. However, several of the *in vitro* findings appeared to relate to *in vivo* outcomes, providing important information for candidate selection. These results demonstrated that NEs can be produced with a wide variety of adjuvant activities based on formulation, and suggest the possibility of optimized surfactant combinations for specific applications.

## Materials and Methods

### Cell Lines

Murine epithelial, dendritic and macrophage cell lines were purchased from American Type Culture Collection, Manassas, VA and demonstrated to be free of mycoplasma contamination by RT-PCR (PCR Mycoplasma Detection Set, Takara Bio Inc.). Cell lines used included TC-1 (epithelial), Jaws II (dendritic), and Raw 264.7 (macrophage). For NF-κB reporter assays Raw-blue (macrophage) cells used were purchased from Invivogen, San Diego, CA. TC-1 (ATCC, CRL-2785) is a murine epithelial cell line derived from lung epithelial cells from a C57BL/6 mouse. JAWS II (ATCC, CRL-11904) is an immortalized immature myeloid-type dendritic cell line derived from a p53-deficient C57BL/6 (H-2^b^) mouse. Raw 264.7 (ATCC, TIB-71) is a macrophage cell line established from the ascites of an Abelson murine leukemia virus (A-MuLV)-induced tumor from a BAB/14 mouse, and Raw-blue is a macrophage reporter cell line derived from Raw 264.7 and expresses secreted embryonic alkaline phosphatase (SEAP) gene under the control of inducible NF-κB and AP-1 transcription factors.

TC-1 cells were grown in RPMI 1640 media with L-glutamine (Corning), containing 10% heat-inactivated FBS (HI-FBS) (Gemini), 1 x nonessential amino acids, 10 mM HEPES, 100 IU penicillin, and 100 μg/mL streptomycin (Gibco). Jaws II cells were maintained in MEM-α media containing 10% HI-FBS, 2 mM L-glutamine, 1 mM sodium pyruvate, 5 ng/mL mouse granulocyte macrophage colony-stimulating factor (mGM-CSF), 100 IU penicillin, and 100 μg/mL streptomycin. Raw 264.7 cells were maintained in DMEM 10% FBS supplemented with penicillin/streptomycin (100 IU/ml) (Mediatech). Raw Blue cells were grown in DMEM with 10% HI-FBS, 100 μg/mL Normocin (Invivogen), 200 μg/mL Zeocin and 2 mM L-glutamine.

### Preparation of Nanoemulsions

**Fig 1:**
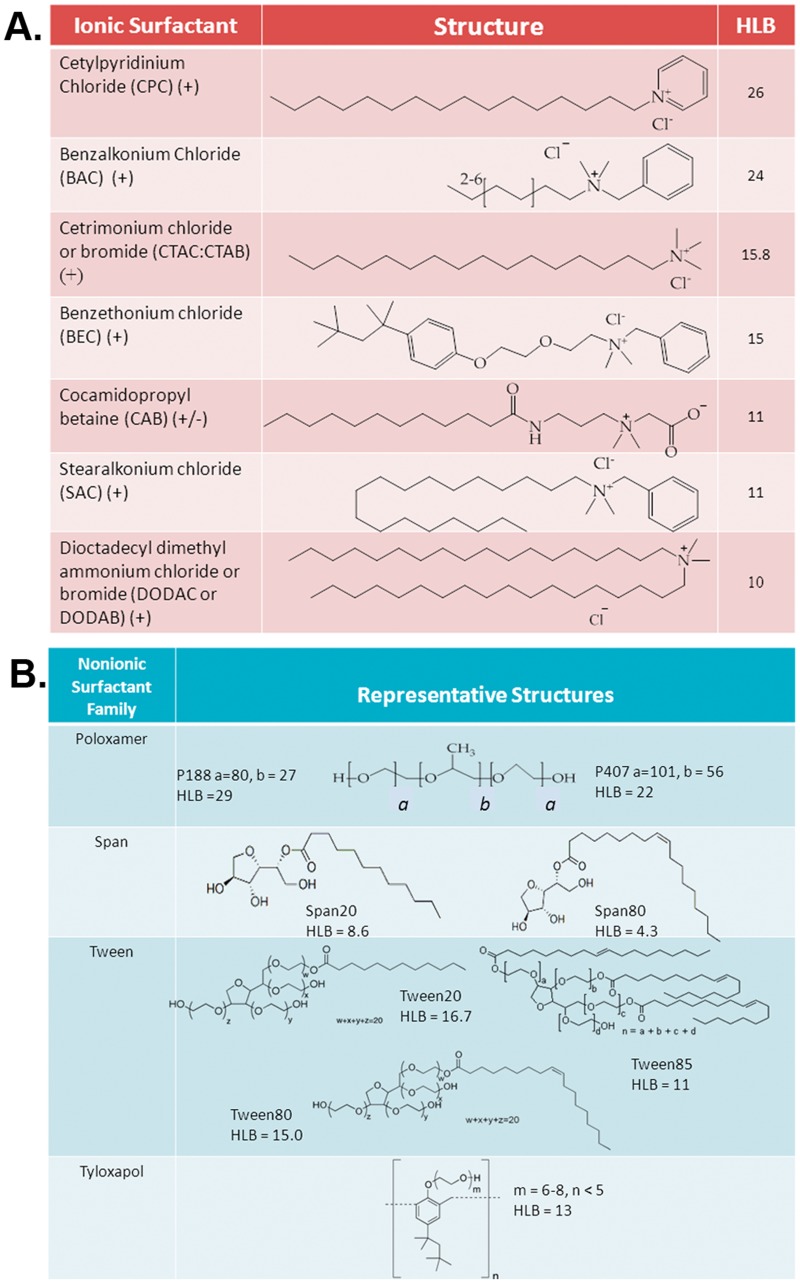
Surfactant chemical structures. **(A)** Chemical structures, abbreviations and HLB values of ionic and (B) nonionic surfactants used to formulate NEs.

NE formulations were provided by NanoBio Corporation, Ann Arbor, MI. Briefly, NEs were manufactured by high-speed emulsification of ionic and nonionic surfactants, ethanol (200 proof), soybean oil and purified water using a high speed homogenizer. A series of NEs with combinations of various ionic and nonionic surfactants at different ratios were produced. Ionic surfactants were selected based on the differences in the structures of the polar head groups and hydrophobic tails in order to produce NEs with diverse physicochemical properties. All cationic surfactants were quaternary ammonium surfactants with a +1 charge. Surfactant structures and abbreviations as well as their hydrophilic-lipophilic balance values (HLB) are shown in Fig 1A and 1B. Only NEs that were deemed stable, (no changes in physical appearance, particle size distribution, or zeta potential over 2 weeks at 22°C and 40°C) were used for these studies. Surfactant blend ratios of the NEs are annotated according to the ratio of ionic to nonionic surfactants. For example, a CPC/Tween80 NE annotated as 1:6 contains one part CPC to six parts Tween80 (by weight), and a NE of Tween80 with no cationic surfactant is annotated as 0:6.

### High Throughput Cellular Toxicity Screening

Cellular toxicity was evaluated using an automated format of the CellTiter-Glo Luminescent Cell Viability Assay (Promega) at the High-Throughput Screening Laboratory (Life Sciences Institute, University of Michigan). Cells were seeded overnight on 384-well plates at 37°C. Cell suspension was dispensed using a Multidrop 384 (Thermo Scientific) system. Seeding densities for different cell types were as follows: TC-1: 1 x 10^4^ cells/well; Jaws II: 1.5 x 10^4^ cells/well; RawBlue: 2.0 x 10^4^ cells/well in 40 μL of media/well. 3-fold serial dilutions of NE were prepared in the respective cell culture medium, spanning a 100,000-fold concentration range (1–0.000017% NE (w/v)). Media was removed from the plates, and 40 μL of the NE dilutions were added to each well. Each condition was run in duplicate. Cells were incubated for 24 h at 37°C. Supernatant was aspirated with an ELx405 microplate washer (Biotek), and cells were washed with PBS (3x). 10 μL of CellTiter-Glo reagent was added to each well and incubated at RT for 15 min. Luminescence was measured on a PHERAstar plate reader (BMG LabTech). The IC50 was defined as the NE concentration (% w/v) at which there is 50% cell viability after 24 h of treatment.

### Antigen uptake

To measure antigen uptake, TC-1 cells were seeded overnight in 24-well plates at a density of 1 x 10^5^ cells per well in TC-1 culture media. A 10 times concentrated mixture of NE (0.25%, 0.5%, 1%) and DQ-Ovalbumin (50 μg/mL) (DQ-OVA) (Life Technologies) was prepared in PBS, pH 7.4, and incubated for 15 min at room temperature (RT). DQ-OVA is ovalbumin conjugated to a self-quenching fluorophore. DQ-OVA is minimally fluorescent while OVA is intact, but upon proteolytic processing of OVA, the fluorophore is unquenched, resulting in a large increase in fluorescence. Cell culture media on the cells was replaced with fresh TC-1 media, and the NE-OVA solution was then added to the media to obtain final concentrations of 0.025%, 0.05%, and 0.1% NE with 5 μg/mL DQ-OVA. Cells were treated for 2h at 37°C. Media was collected from each well and placed in separate flow cytometry tubes. The wells were rinsed with 1 mL of PBS, and this wash was collected and added to the corresponding tubes. The remaining adherent cells in each well were then removed by trypsinization, and added to the corresponding flow cytometry tubes containing the media collected from the wells and the PBS wash. Wells were rinsed with an additional 1 mL of PBS to ensure collection of all remaining cells, and this wash was collected and added to the corresponding tubes. Samples were spun at 2000 rpm for 5 min at 4°C. The supernatant was discarded, and the cells were washed with 4 mL of FACS buffer (PBS containing 0.1% bovine serum albumin and 0.1% sodium azide). Cells were spun again, and the pellet was resuspended in 500 μL of FACS buffer. Flow cytometry was performed on a Beckman Epics XL flow cytometer. The mean fluorescence intensity (MFI) for cells with DQ-OVA uptake (and proteolytic processing) was measured on FL-1 (excitation 488 nm, emission 530/30 nm). Uptake for Jaws II and Raw cells were performed similarly.

### Uptake by Confocal Microscopy

TC-1 cells were seeded on 8-well chambered coverglass slides (Lab-Tek) overnight at 37°C. Media was replaced with fresh media containing 0.05% NE with 10 μg/mL DQ-OVA. Cells were incubated at 37°C for 2 h, and then washed 2x with PBS, fixed in 4% paraformaldehyde, allowed to dry before addition of ProLong Gold with DAPI (Life Technologies). Cells were imaged on an LSM 510-META laser scanning confocal fluorescence microscope (Zeiss) (green fluorescence ex = 488 nm em = 500–530 nm was measured for DQ-OVA).

### NF-κB Activation

NF-κB activation was measured using Raw-Blue cells with the Quanti-Blue assay (Invivogen). SEAP activity was quantified by the Quanti-Blue colorimetric substrate according to the manufacturer’s protocol. Briefly, 100,000 cells were mixed with NEs in 200 μL of media without zeocin in 96-well plates over a concentration range of 5x10^-5^-1% in media. As the positive control, lipopolysaccharide (LPS) was mixed with cells over a concentration range of 1.0 x 10^-6^-1 μg/mL. Cells were incubated at 37°C for 24 h. At the end of the incubation, 20 μL of each supernatant was transferred to a new 96-well plate, and 200 μL of Quanti-blue reagent was added per well. Plates were incubated at 37°C for 24 h, and SEAP levels were determined by measuring the absorbance at 650 nm. A blank plate was prepared with NE dilutions alone in Quanti-Blue, and the absorbance was subtracted from the corresponding supernatant sample to correct for interference due to NE turbidity. Results are shown as the average of duplicates. After the NE treatment, cells were rinsed once with PBS, and viability was assessed by an XTT assay according to the manufacturer’s protocol (Roche). SEAP levels were normalized to viable cells left after 24 h of treatment.

### Mucoadhesion

Dynamic light scattering (DLS) and zeta potential (ZP) measurements were performed consecutively for the same sample on a Zetasizer Nano-ZS (Malvern Instruments Ltd). Porcine gastric mucin type III (mixture of different mucin isoforms) (Sigma-Aldrich), was rehydrated at 1 mg/mL in 1 mM HEPES pH 7 at RT for 30 min prior to performing the assay. 0.1% NE (w/v) was mixed with 0.05 mg/mL mucin in 1 mM HEPES pH 7, and incubated for 2 m before measurements. Particle size (PS) is expressed as average diameter (Z_ave_d). ΔZave represents the difference between the PS with mucin (Z_ave final_) and the PS without (Z_ave init_). ΔZP represents the difference between the ZP without mucin and the ZP with mucin (ZP_init_—ZP_final_).

### Animals

Pathogen-free, 8-week-old female C57BL/6 mice (Charles River Laboratories) were housed in specific pathogen-free conditions. All procedures were approved by the University Committee on the Use and Care of Animals (UCUCA) at the University of Michigan and were performed in accordance with these guidelines.

### Immunization and Acute Response

For acute cytokine response immunization studies, mixtures of NE and ovalbumin (NE-OVA) at final concentrations of 20% NE (w/v) and 1.33 mg/mL OVA (endograde ovalbumin, Hyglos GmbH) in PBS, pH 7.4 were prepared. NE-OVA formulations were vortexed, and incubated for at least 15 min at RT prior to immunization. C57BL/6 mice (n = 4 per treatment group) were immunized IN with 15 μL (7.5 μL/nare) of the NE-OVA mixture (20% NE and 20 μg OVA/mouse) or with OVA alone in PBS. NE formulations evaluated were CPC/W80 1:6, DODAC/W80 1:6, DODAC/P188 1:6, CTAC/P188 1:6, DODAC/W80 1:1, CPC/P188 1:6, DODAC/P188 6:1, DODAC/P407 1:1, CAB/W80 1:6. Mice were sacrificed 22h after immunization. Bronchial alveolar lavage (BAL) fluid was obtained as previously described [8], and the nasal septum was harvested from immunized mice. Nasal septa (NS) were homogenized in 350 μL of T-PER tissue extraction buffer (Thermo Scientific), and frozen at -80°C. Samples were subjected to an additional freeze/thaw cycle, and then centrifuged at 10,000xg for 5 min at 4°C to remove debris. Supernatants were saved. Total protein concentrations in the NS and BAL were quantified using a BCA assay (Thermoscientific). BAL samples and nasal septum supernatants were analyzed using a Milliplex MAP 8-plex magnetic multiplex kit with a mouse cytokine/chemokine panel (Millipore) customized for G-CSF, IFN-γ, IL-5, IL-6, IL-9, IL-13, IL-17, and TNF-α according to the manufacturer’s protocol. Cytokine levels were normalized to total protein concentration.

### Immunization and Humoral Response

For humoral response immunization studies, NE-OVA mixtures with final concentrations of 20% NE (w/v) and 1.3 mg/mL OVA (endograde ovalbumin, Hyglos GmbH) in PBS, pH 7.4 were prepared. NE-OVA formulations were vortexed, and incubated for at least 15 min at RT prior to immunization. C57BL/6 mice (n = 5 per treatment group) were immunized IN with two administrations, 4 weeks apart, of 15 μL (7.5 μL/nare) of the NE-OVA mixture (20% NE and 20 μg OVA/mouse). This dosage of NE and OVA was determined to be optimal in our previous studies [19, 20]. NE formulations evaluated included CPC/W80 1:6, DODAC/W80 1:6, DODAC/P188 1:6, CTAC/P188 1:6, DODAC/W80 1:1, CPC/P188 1:6, DODAC/P188 6:1, DODAC/P407 1:1, CAB/W80 1:6. DODAC/W80 1:6 mice were immunized separately from the other NEs listed. However, CPC/W80 1:6 run as an internal control gave consistent results between the two studies. Serum was obtained from the saphenous vein every 2-weeks post-initial immunization, and anti-OVA specific IgG end-titers were measured by ELISA as previously described [8]. Briefly, serum samples were serially diluted in PBS with 0.1% BSA, and incubated on microtiter plates coated with 20 μg/mL OVA. ELISAs were developed with an alkaline phosphatase detection system, and quantified by measuring the optical density (OD) at 405 nm (OD_405_). Endpoint titers are reported as the reciprocal of the highest serum dilution giving an OD above a cutoff value (sum of OD of the same dilution of a control serum from an untreated mouse and two times the standard deviation).

Animals were sacrificed 10 weeks post-initial immunization. BAL samples were collected as above, and spleens were harvested and mechanically disrupted in PBS to obtain single-cell suspensions for cytokine response measurements. Splenocytes were spun at 2000 rpm for 5 min, and resuspended in ACK lysis buffer for <3m to remove red blood cells (150 mM NH_4_Cl, 10 mM KHCO_3_, 0.1 mM EDTA). PBS was added to stop lysis, and cells were spun and washed again in PBS. The cell pellet was then resuspended in T-Cell media (DMEM medium supplemented with 5% FBS, 2 mM L-glutamine, 1 x nonessential amino acids, 1 mM sodium pyruvate, 10 mM MOPS, 50 μM 2-mercaptoethanol, 100 IU penicillin, and 100 μg/mL streptomycin and filtered through a cell strainer. Splenocytes were then plated at a density of 4 x 10^5^ cells/well in 96-well tissue culture plates and stimulated as below.

### Cellular Recall Response

Cellular response was evaluated at sacrifice (5 weeks post boost) in splenocytes isolated as described above. Isolated splenocytes on 96-well plates were stimulated with 20 μg/mL ovalbumin (Hyglos, GmbH) in 100 μL of media for 48 h at 37°C. Cell supernatant was collected, and cytokine levels were assessed using a Milliplex MAP mouse cytokine/chemokine magnetic multiplex kit (Millipore) customized for IFN-γ, IL-2, IL-5, IL-6, IL-10, IL-17 and IL-13 following the manufacturer’s protocol. Supernatants from DODAC/Tween80 1:6 mice were run on a Milliplex MAP mouse cytokine/chemokine polystyrene multiplex kit (Millipore)

## Results

### NE formulation and Physical Characterization

NEs are oil-in-water emulsions consisting of an oil (highly refined soybean oil), a solvent (ethanol), and water emulsified with a mixture of quaternary amine cationic (or zwitterionic) and nonionic surfactants. We generated a large series of NE formulations by using a variety of cationic and nonionic surfactants combined at various ratios. After >200 formulations were manufactured, the 112 NE’s that met long-term stability criteria (as defined in the methods) were included in this study.

A series of NEs were produced using the cationic surfactant CPC in combination with various nonionic surfactants, as our prior work demonstrated strong adjuvant activity for a CPC/Tween80 formulation that was dependent on the presence of CPC [8, 11, 13]. Furthermore, CPC has already been FDA approved for use in commercial healthcare products. CPC consists of a single C16 hydrophobic tail coupled with a pyridinium ring polar head group, giving it a +1 charge. It has a relatively high hydrophilic-lipophilic balance (HLB) value of 26, making it the most hydrophilic surfactant used in the NE library. The majority of NEs containing CPC in combination with various nonionic surfactants were found to be stable at the surfactant blend ratios tested. Other series of NEs were made with different cationic surfactants including benzalkonium chloride (BAC), cetrimonium chloride/bromide (CTAC/CTAB), stearalkonium chloride (SAC), benzethonium chloride (BEC), and dioctadecyl dimethyl ammonium chloride/bromide (DODAC/DODAB). (Fig 1A). These cationic surfactants were chosen for their diverse physicochemical properties. BAC was chosen as it has a larger polar head group than CPC and shorter tail length (C6–C10). CTAC/CTAB, in contrast, has a much smaller polar head group than CPC and BAC coupled to the same C16 hydrophobic tail as CPC, making it more hydrophobic (HLB 15.8) than CPC, and perhaps less prone to migration into the aqueous phase. SAC was chosen as it has the same head group as BAC, but coupled to a longer hydrocarbon tail (C18) than both CPC and BAC. BEC was included in order to investigate the effect of a larger surfactant polar head group with a small (C4) branched hydrocarbon tail, and DODAC/DODAB was included to look at the effect of having an additional hydrophobic chain on the cationic surfactant. DODAC/DODAB consists of two C18 hydrophobic tails and has a small polar head group similar in size to that of CTAC. Lastly, the zwitterionic surfactant, cocamidopropyl betaine (CAB) was also included as it has a C11 tail with a large polar head group that carries both a positive and negative charge due to the betaine group. Nonionic co-surfactants were chosen from four different families; Poloxamers, Spans, Tweens, and Tyloxapols, for their HLB values which suggested they would form stable NEs with the selected cationic surfactants (Fig 1B).

**Fig 2:**
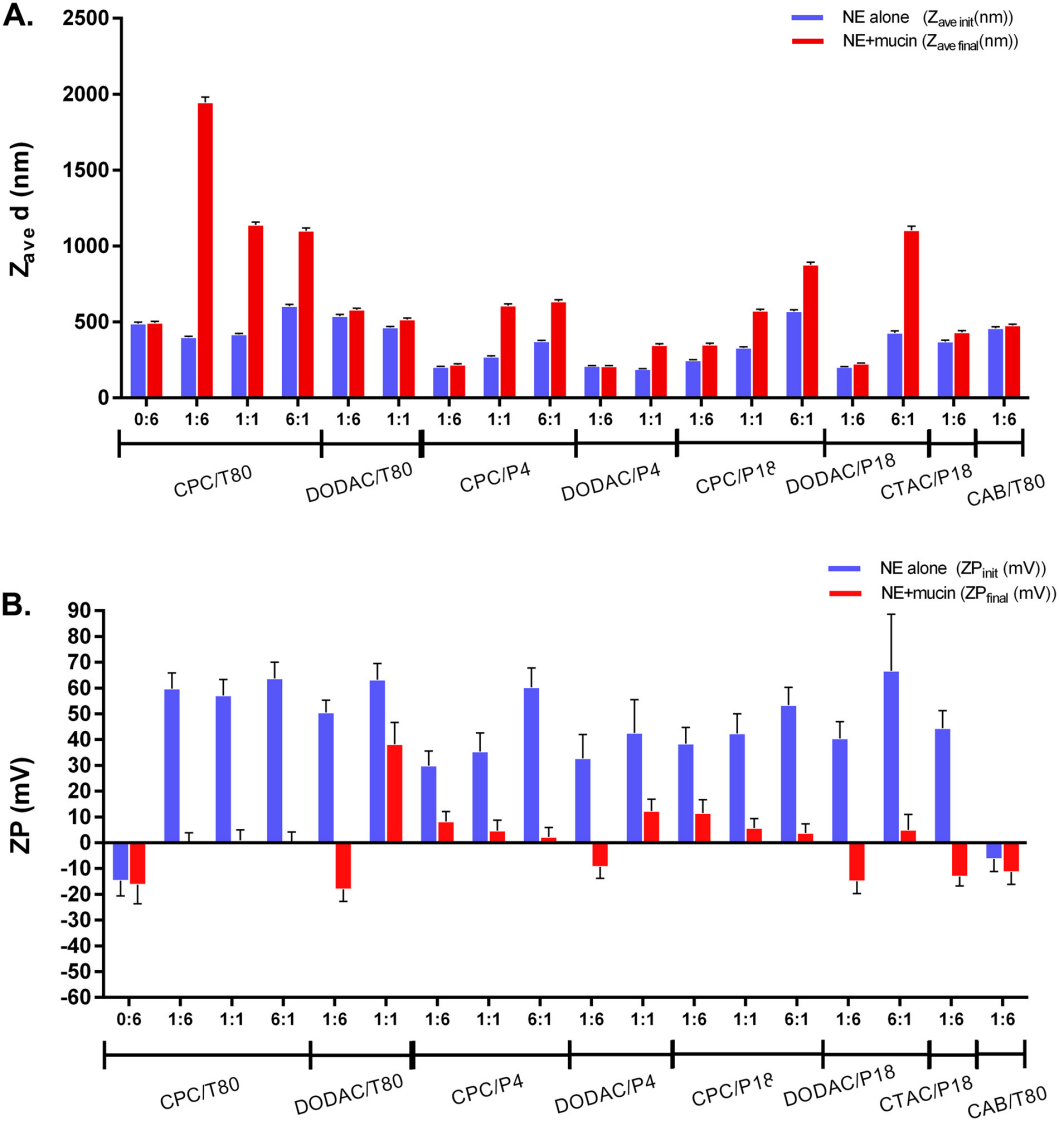
Mucoadhesion of NE. (A) Particle size (Zave d) and (B) ZP of 0.1% NE incubated with and without 0.05 mg/mL mucin.

The particle size distributions of the NEs were determined by DLS, and the average particle diameters (Z_ave_) are shown in S1 Table. NE size distributions were unimodal with low polydispersity (PdI< 0.2); however, the average droplet size varied significantly based on the surfactant composition, ranging from 250 to 800 nm. For example, CPC/P407 1:6 had a Z_ave_ of 202 nm, while CPC/Tween80 1:6 had a Z_ave_ of 406 nm even though the formulations contained the same amount of CPC and nonionic surfactant. The NE zeta potential (ZP) was also determined and values ranged from -12 mV to 75 mV, dependent primarily on the cationic:nonionic ratio. Increasing the cationic to nonionic ratio also increased the NE droplet surface charge (ZP_init_) as expected (S2 Table). For example, increasing the CPC/Tween80 ratio from 0:6 to 1:6 increased the ZP_init_ from -14.7±5.9 mV to 59.8 ±6.1 mV, and further increasing the CPC to a 6:1 ratio gave a ZP_init_ of 63.7±6.3 mV. Particle sizes and charges for representative NEs are shown in Fig 2A.

### *In Vitro* Screening Studies

#### Mucoadhesion

Several studies have demonstrated that mucoadhesion and the depot effect significantly influence mucosal adjuvant effectiveness [23, 28–30]. We have previously demonstrated that a strong association of NE adjuvant with the mucus protein mucin, *in vitro*, corresponds to improved antigen retention in the nasal mucosa and a greater uptake of antigen-adjuvant through the mucous layer *in vivo* [19]. In contrast, an absence of NE association with mucin *in vitro* corresponded to significantly reduced antigen uptake and immunogenicity. Therefore, given the apparent importance of adhesion, the associative properties of the NE formulations in the library with mucin were screened *in vitro*.

NE-mucin interaction was determined by characterizing the change in NE particle size and surface charge before and after mucin addition by DLS and ZP measurements, respectively. Association with mucin is reflected by an increase in particle size (ΔZ_ave_) and a decrease in surface charge (ΔZP) due to the negative charge of mucin. Initial and final Z_ave_ and ZP values are shown in Fig 2, and corresponding ΔZave and ΔZP values are shown in S1 Fig. Data for representative NEs are highlighted in Fig 2, while the results for all formulations are summarized in 2D representations in S1 Fig and in S1 and S2 Tables. A positive surface charge appeared to be critical for mucin association. Little or no mucin association was observed for the zwitterionic NE CAB/Tween80 1:6 and the neutral NE, Tween80 0:6. In contrast, significant association could be obtained by increasing the CPC in CPC/Tween80 to ratios of 1:6, 1:1, and 6:1, as reflected by the large ΔZ_ave_ values (ex. 1546 ± 36 nm for 1:6) (Fig 2A and S1 Fig). Binding of mucin to cationic NEs decreased the ZPs, presumably because the negatively charged protein neutralizes the positive surface charge (Fig 2B and S1 Fig). The ZP of Tween80 0:6 and CAB/Tween80 1:6, however, did not change significantly upon addition of mucin, again indicating little mucin binding. Similar trends of larger ΔZ_ave_ and ΔZP values with increasing cationic:nonionic ratios were observed with most of the surfactant families evaluated (S1 Fig).

As shown in Fig 2A and S1 Fig, CPC NEs had larger ΔZ_ave_ values compared to DODAC and CTAC NEs when combined with identical nonionic surfactants at the 1:6 ratio. For example, CPC/Tween80 1:6 and DODAC/Tween80 1:6 both had similar Z_ave init_ and ZP_init_ values, and also showed similar magnitudes of ΔZP; however, CPC/Tween80 1:6 displayed larger changes in particle size than DODAC/Tween80 1:6 (ΔZ_ave_ = 1546 nm vs. 41.1 nm). These results suggest that there are different types of interactions between the NE and mucin that are dependent on the surfactant composition of the NE. Of interest, scatter plots of ΔZ_ave_ and ΔZP vs ZP_init_ demonstrated that while positive surface charge is critical for an interaction with mucin, it is not the sole determinant of the degree and type of interaction. Three different types of NE-mucin interactions were observed: those with large ΔZP and large ΔZ_ave_ (binding and aggregation) those with large ΔZP but small or no ΔZ_ave_ (binding without aggregation), and those with small or no ΔZP or ΔZ_ave_ (no binding) (S1 Fig). While the implications of these findings were not fully understood, the clear division between the types was useful during the selection process used for identifying which NEs to test *in vivo* for adjuvant activity.

#### High Throughput Screening of NE Enhancement of Cellular Antigen Uptake

Antigen uptake by epithelial and antigen presenting cells is critical for antigen processing and presentation. Given the importance of uptake in epithelial cells, compounds in the NE library were evaluated for their ability to enhance antigen uptake in the TC-1 mouse lung epithelial cell line. To distinguish intracellular uptake from simple adhesion to the cell surface, self-quenched fluorescently labeled OVA (DQ-OVA) was used as the antigen. DQ-OVA fluorescence remains quenched until the antigen undergoes proteolytic processing. Thus, an increase in cellular fluorescence, as measured by flow cytometry, corresponds to both intracellular uptake and processing of OVA.

**Fig 3:**
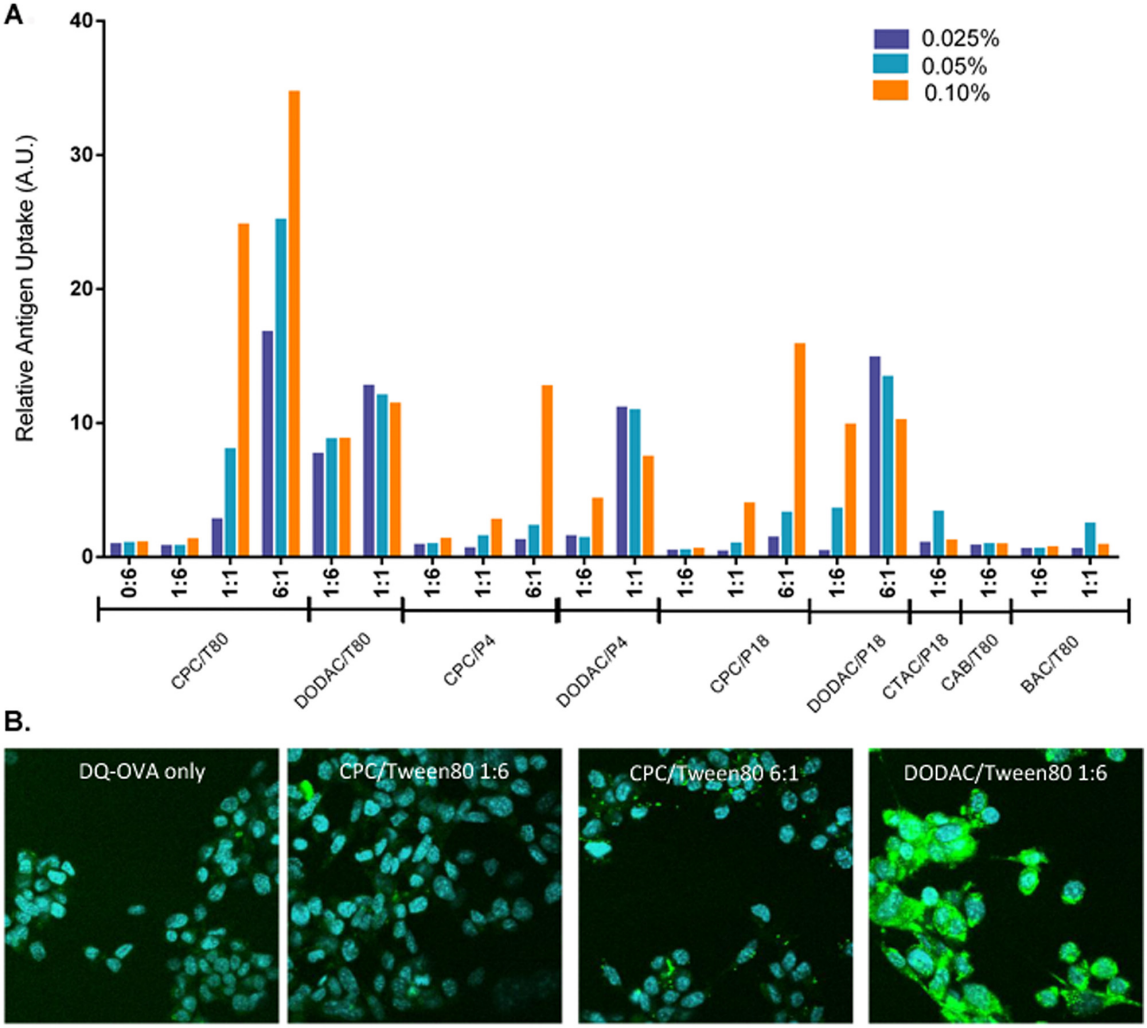
NE facilitated uptake of DQ-OVA in TC-1 epithelial cells. **(A)** TC-1 cells were incubated with the indicated NE concentrations and DQ-OVA for 2 h at 37°C. Cellular fluorescence was measured by flow cytometry. Relative antigen uptake is expressed as fold fluorescence (MFI at ex 488 nm, em 533 nm) increase relative to that of cells treated with DQ-OVA alone. Representative NE formulations with increasing cationic:nonionic surfactant ratios are shown. **(B)** Confocal fluorescence microscopy of TC-1 cells incubated with 10 μg/mL of DQ-OVA alone or with 0.05% of the indicated NE for 2 h at 37°C. Nuclei were stained with DAPI (blue), intracellular and proteolytically processed DQ-OVA (green).

NE, over a range of concentrations [0.003125–0.1% (w/v)], was mixed with DQ-OVA (5 μg/mL) and incubated with TC-1 cells for 2 h at 37°C. Uptake enhancement (relative antigen uptake) is expressed as the fold increase of cellular fluorescence relative to that of cells treated with DQ-OVA alone, and a value of 1 represents no enhancement compared to antigen only. The optimal NE concentration range for uptake enhancement was found to be between 0.025–0.1% (w/v). Uptake levels are shown at this concentration range for representative NEs (Fig 3). The entire set of NE formulations in the library was subsequently evaluated and the results are shown below in various 2D scatter plot representations versus cytotoxicity in the following section, and in the S3 Table (for the 0.1% NE treatment concentration). The intracellular location of antigen was confirmed by confocal fluorescence microscopy (Fig 3B).

Not surprisingly, low intracellular fluorescence was observed for cells treated with DQ-OVA alone; however combining DQ-OVA with several NE formulations markedly increased the fluorescence (Fig 3). NE-facilitated DQ-OVA uptake showed a large range in the degree of enhancement (from no enhancement to >30 fold enhancement) depending on the surfactant composition, the cationic:nonionic ratio, as well as the NE concentration. CPC containing NEs with a cationic:nonionic ratio of 6:1 gave the greatest enhancement of all the NEs tested regardless of the co-surfactant as shown in Fig 3 and in the 2D scatter plot representations discussed in the following section. Enhancement of uptake with NEs containing the same nonionic surfactant decreased when the CPC content was reduced from a 6:1 ratio to a 1:1 ratio, and again upon reduction to a 1:6 ratio. Additionally, while all of the 1:1 CPC NEs tested showed uptake enhancement, only a few 1:6 CPC NEs showed uptake enhancement and it was dependent on the nonionic surfactant in these formulations. For example, while CPC/Tween80 1:6 had a relative antigen uptake value of 1.4, CPC/P188 1:6 actually showed reduced uptake as compared to antigen alone (Fig 3). Scatter plots of uptake vs. ZP demonstrate a positive correlation between cationic surface charge and antigen uptake enhancement for the CPC NEs as well as the other cationic surfactants as discussed below in the 3D scatter analysis section below. For example, increasing the DODAC/P407 ratio from 1:6 to 1:1 (corresponding to an increase in ZP from 32.7 to 42.6 mV) increased the relative antigen uptake from 4.4 to 7.6 at 0.1% NE. In contrast, mixing DQ-OVA with either the zwitterionic CAB/Tween80 1:6 (ZP of -6.25±4.9 mV) or CAB/P407 1:6 (ZP of -2.07±7.7 mV) gave no significant enhancement. These results suggested that a net positive charge is necessary for NE enhancement of antigen uptake.

Of interest, at the lower cationic:nonionic ratios, NEs with some non-CPC cationic surfactants gave better enhancement of uptake. DODAC/DODAB NEs enhanced antigen uptake to a greater extent than the corresponding CPC NEs with the same nonionic surfactant at the 1:6 ratio. DODAC/Tween80 1:6 had a relative antigen uptake of 6.2 compared to 1.4 for CPC/Tween80 1:6 at 0.1% NE. Even at 0.025% NE, DODAC/Tween80 1:6 induced similar uptake as CPC/Tween80 1:1 at double the NE concentration (Fig 3). However, increasing the CPC in CPC/Tween80 to 1:1 increased the uptake to levels >2 times that of DODAC/Tween80 1:1 at the 0.1% NE concentration, and further increasing the CPC to CPC/Tween80 6:1 gave uptake >3 times that of NEs with DODAC at 1:1 and 6:1 ratios. Thus, while less DODAC content is required to enhance epithelial cell antigen uptake, at the higher concentrations CPC NEs are more effective. This is despite the fact that DODAC/Tween80 1:6 and CPC/Tween80 1:6 both have similar particle sizes and ZPs, suggesting that uptake is not dependent on charge and particle size alone. Similar trends were observed with CPC and DODAC formulations combined with other nonionic surfactants as illustrated by the 2D scatter plots discussed in the following section and in the S3 Table. For instance, at 0.1% NE, DODAC/P188 1:6 induced 10-fold greater uptake than CPC/P188 1:6. Furthermore, DODAC/P407 1:1 at 0.025% induced the same amount of antigen uptake as CPC/P407 6:1 at 0.1%. Lastly, upon examining the uptake of DQ-OVA by confocal microscopy, a clear difference in the antigen localization between CPC/Tween80 and DODAC/Tween80 treated cells could be observed (Fig 3B). CPC/Tween80 1:6 and 6:1 treated cells had very punctate areas of intense fluorescence in a percentage of the cells, whereas DODAC/Tween80 1:6 showed a much more uniform intracellular distribution of DQ-OVA within a larger percentage of cells. This could reflect differences in the uptake mechanisms and antigen processing pathways for these different cationic surfactants. Thus, while CPC/Tween80 6:1 showed nearly three-fold higher relative antigen uptake than DODAC/Tween80 1:6 by flow cytometry, this is likely due to the higher mean fluorescence intensity found on the percentage of cells that took up DQ-OVA, even though a greater number of DODAC/Tween80 1:6 treated cells were fluorescent, they had a lower mean fluorescent intensity. Furthermore, as confocal images were only examined for adherent cells, it does not account for the cytotoxicity of CPC/Tween80 6:1 (as discussed below) compared to the non-cytotoxic DODAC/Tween80 1:6. Cells which take up very large amounts of CPC/Tween80 6:1 and DQ-OVA that account for the highest relative antigen uptake values observed by flow cytometry for this NE are likely killed and washed off before imaging.

Finally, while the concentration and type of cationic surfactant predominantly determines antigen uptake, the nonionic surfactant does appear to play a role in this process. For example, at 0.025% NE, DODAC/Tween80 1:6 induced 7-fold greater uptake than DODAC/P188 1:6. The concentration of DODAC/P188 1:6 had to be raised to 0.1% to achieve a comparable level of uptake as 0.025% DODAC/Tween80 1:6. Therefore, while the cationic surfactant type and blend ratio are the dominant factors in the uptake properties of the NE, the activities are fine-tuned by the nonionic surfactant.

In contrast to the TC-1 cells, none of the formulations significantly enhanced antigen uptake in Jaws II (DCs) or RawBlue (macrophages) relative to antigen alone. These cells took up a very large amount of antigen even in the absence of NE (data not shown).

#### High Throughput Cellular Toxicity Screening

The induction of a moderate degree of cytotoxicity and/or irritation by pathogens or adjuvants can lead to danger signals that activate innate immunity and inflammation [21, 22, 31–33]. We thus screened the NE library for cytotoxicity with three different mouse cell line types: epithelial cells (TC-1), dendritic cells (Jaws II), and macrophages (Raw-Blue). Toxicity was evaluated over a 100,000-fold range of NE concentrations, and the 50% inhibitory concentration (IC50) for each formulation was identified after 24 h of NE exposure using a CellTiter-Glo luminescent cell viability assay.

**Fig 4:**
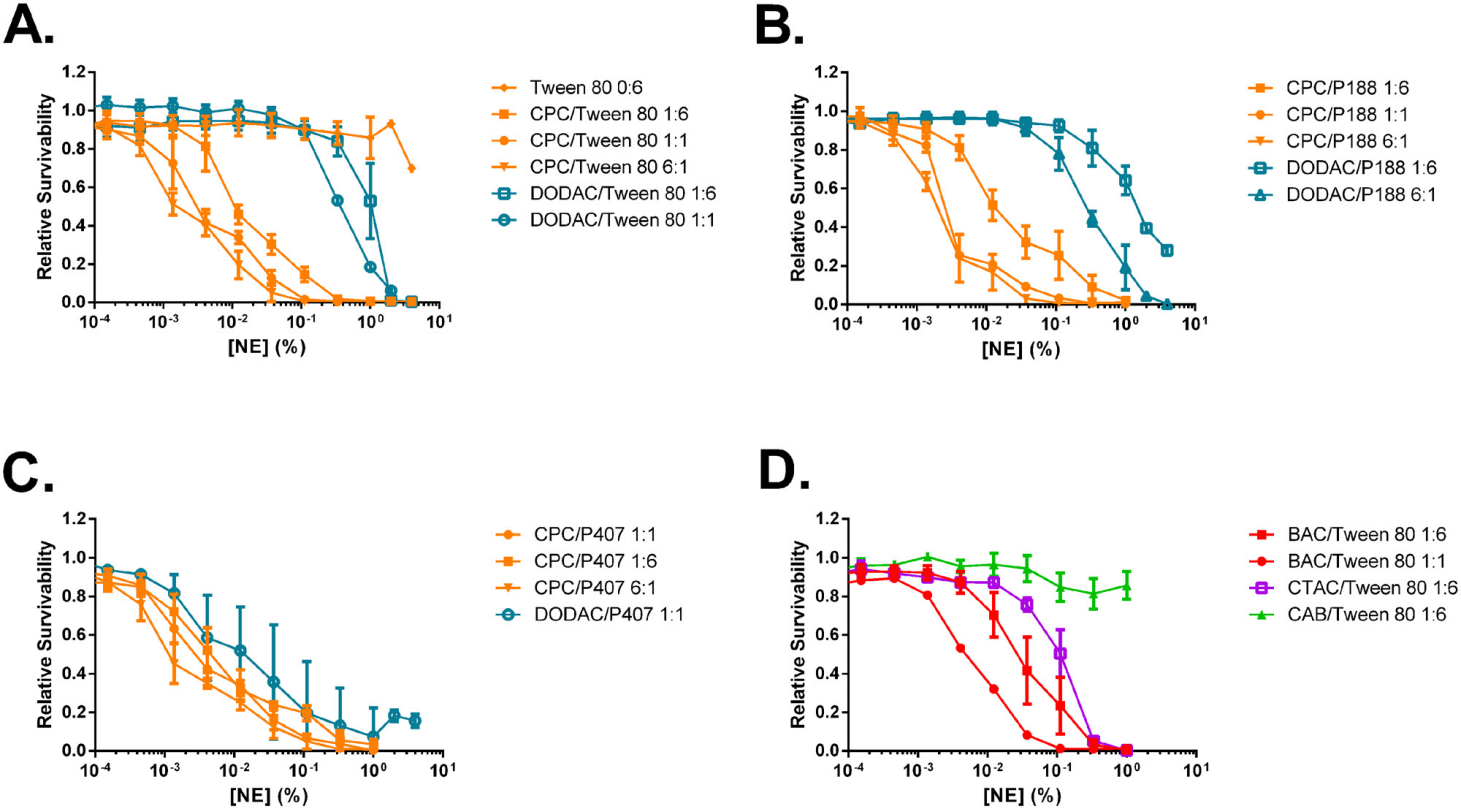
NE induced cytotoxicity for TC-1 cells. Cells were treated for 24 h with increasing concentrations of NE at 37°C. Cytotoxicity was measured with a CellTiter-Glo luminescent cell viability assay. Data for representative NEs grouped by nonionic surfactant are shown. Viability curves for NEs containing (A) Tween80, (B) P188, and (C) P407 with CPC or DODAC cationic surfactants, and for NEs containing (D) Tween80 with BAC, CTAC, and CAB. Curves shown are the average of two independent experiments ± SD.

NE induced cytotoxicity increased as the cationic:nonionic surfactant ratio was increased, and displayed a positive correlation with surface charge for NEs containing the same cationic surfactant. Within groups of NEs containing the same cationic and nonionic surfactant pairs, those with more cationic surfactant gave greater toxicity in all the cell types studied (Fig 4, S2 Fig, and S4 Table). For example, Tween80 0:6 which had no cationic surfactant showed no detectable cytotoxicity in any of the cell types at any tested NE concentration (Fig 4A). As the CPC concentration was increased incrementally in CPC/Tween80 NEs from 1:6, 1:1, to 6:1, the IC50 decreased from 0.017% to 0.0039%, to 0.0023%, respectively in TC-1 cells (Fig 4A), from 0.0076% to 0.0027%, to 0.0014% in Jaws cells, and from 0.0109% to 0.0028% and 0.0020% in Raw cells (S2 Fig). A similar correlation was observed for all of the NEs with cationic surfactants. Zwitterionic CAB/Tween80 1:6 was relatively non-cytotoxic, giving an IC50 of 1.77% in TC-1 cells, supporting the role of cationic charge in cytotoxicity. Cell type had very little impact on the relative cytotoxicity of each NE, and the trends in IC50 values observed in one cell type were maintained in the others (S4 Table).

**Fig 5:**
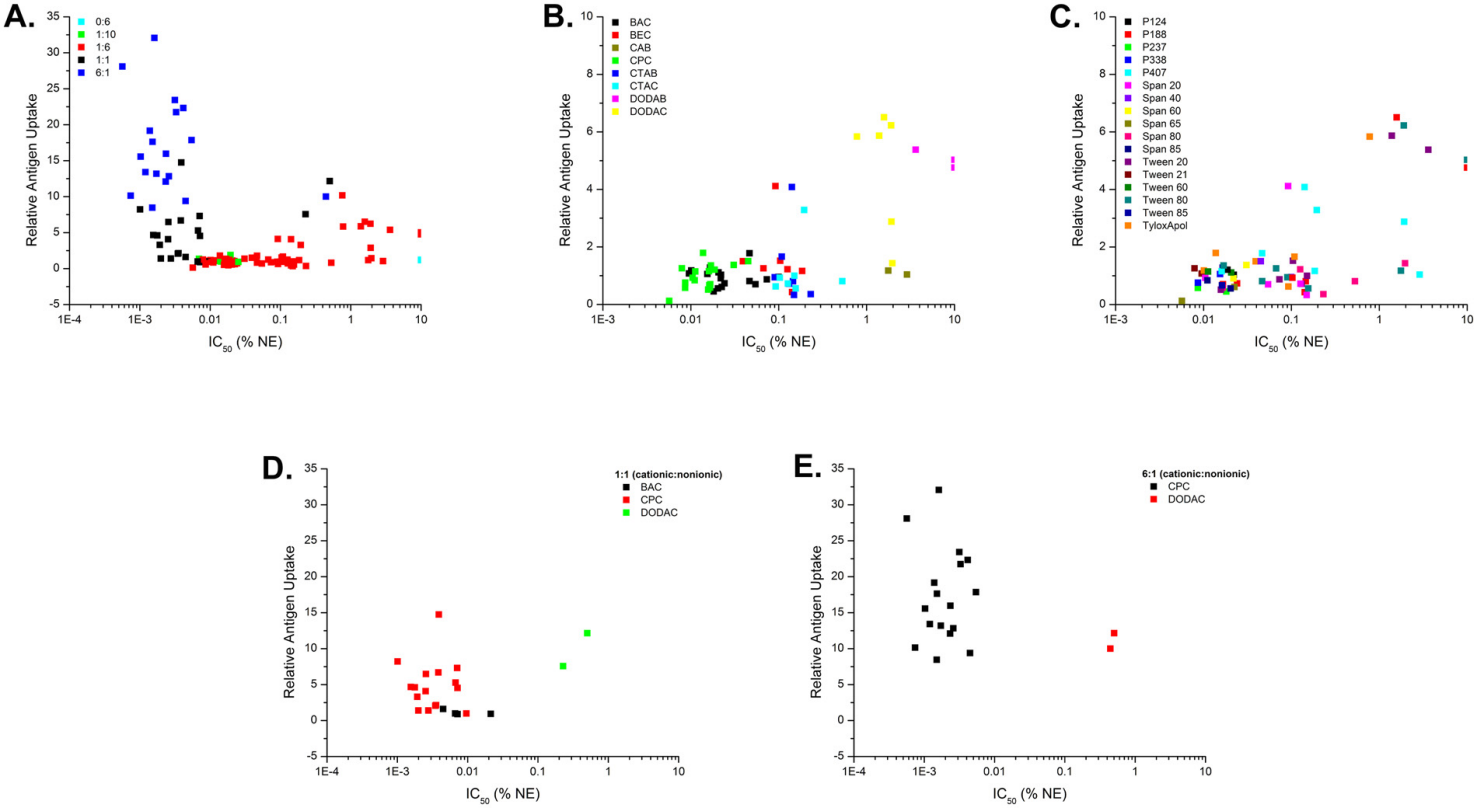
2D scatter plots illustrating the relationship between cytotoxicity (IC50) and antigen uptake. (A) Data points grouped according to cationic:nonionic surfactant ratio for the entire library of NEs. (B) Data points grouped according to cationic surfactant type for the 1:6 NEs. (C) Data points grouped according to nonionic surfactant type for the 1:6 NEs. Data points grouped according to cationic surfactant type for (D) 1:1 and (E) 6:1 NEs.

Of the cationic surfactants studied, CPC formulations exhibited greater cytotoxicity than formulations containing other cationic surfactants in combination with the same nonionic surfactant. For any given nonionic surfactant formulated at a 1:6 cationic:nonionic ratio, the degree of cytotoxicity among the cationic surfactants was CPC > BAC > BEC ≈ CTAC/CTAB >CAB > DODAC/DODAB (Fig 5B and S4 Table). The IC50 values of CPC/Tween80, BAC/Tween80, BEC/Tween80, CTAC/Tween80, CAB/Tween80, and DODAC/Tween80 1:6, for example, were 0.017%, 0.0466%, 0.0671%, 0.156%, 1.77%, and 4.7x10^3^% in TC-1 cells respectively (S4 Table). This same cytotoxicity rank order was observed when these cationic surfactants were combined with other nonionic surfactants as well, such as P188, P407, or Tween20.

Interestingly, NEs containing DODAC as the cationic surfactant demonstrated strikingly reduced toxicity when compared to all other cationic surfactants within each nonionic series. DODAC/Tween80 1:6 had an IC50 of 4.7x10^3^%, compared to 0.017% for CPC/Tween80 1:6 (Fig 3). Increasing the ratio of DODAC to 1:1 and 6:1 increased the cytotoxicity of the NEs; however even at the highest concentration of DODAC (6:1), the NE was still less toxic than the corresponding CPC NEs at the lowest cationic:nonionic ratio (1:10). For example, DODAC/P188 6:1 had an IC50 of 0.4457%, compared to 0.0143% for CPC/P188 1:10 (S4 Table). These findings were surprising, as CPC/Tween80 1:6 had a similar ZP value as DODAC/Tween80 1:6, yet CPC/Tween80 1:6 was much more cytotoxic. Even raising the ZP of the DODAC formulations above that of CPC/Tween80 1:6 by increasing the DODAC ratio to 1:1 and 6:1 still resulted in lower cytotoxicity than the CPC NEs. Thus, while within a group of NEs with the same cationic and nonionic surfactant pair, increasing the charge increases cytotoxicity, cytotoxicity is not dictated by solely by charge and is uniquely related to the surfactant. In general, less effect on cytotoxicity was observed by variation of the nonionic surfactant (Fig 4). While the relative rank order was maintained for the cationic surfactants, a few differences between NEs of the same cationic with a different nonionic surfactant at the same blend ratio were, however, observed. One example was DODAC/P188 1:6, which was much more cytotoxic (IC50 35.15%) than DODAC/Tween80 1:6 (IC50 9.5 x 10^3^%) possibly due to the differences in their particle sizes.

Scatter analysis of antigen uptake versus cytotoxicity (IC50) revealed a strong relationship between toxicity and uptake for non-DODAC NEs, with formulations of greater toxicity (lower IC50) generally displaying higher levels of uptake (Fig 5 and S3 Fig). There appeared to be a threshold effect for non-DODAC NEs, where cationic NEs with an IC50 value above ~0.007% showed relatively similar low levels of antigen uptake (Fig 5A and S3 Fig). However, non-DODAC NEs with IC50s lower than 0.007% showed a substantial increase in antigen uptake as the toxicity increased. While regression analyses gave inadequate correlation fits, demonstrating the complexity of the system (uptake is not dictated by cytotoxicity alone), it is clear that a positive relationship exists between toxicity and uptake for non-DODAC formulations. NEs with CPC (at 1:6 and 1:1) are characterized by comparatively greater cytotoxicity (IC50 < 0.01) than the other cationic surfactants, and gave low or intermediate levels of uptake in epithelial cells. The 6:1 CPC formulations proved to be the most cytotoxic NEs, and also gave the highest levels of uptake overall of all the formulations tested (Fig 5A). NEs with BAC at 1:6 displayed similar cytotoxicity as CPC 1:6 formulations, and also induced similar levels of antigen uptake (Fig 5B). In contrast, as discussed above, DODAC NEs displayed markedly low cytotoxicity (IC50 >> 0.1) and but had the highest uptake of all the NEs at the 1:6 ratio. Notably, maximum uptake (10–15 relative antigen uptake) was observed at NE concentrations >10-30-fold below the IC50 for the DODAC formulations. In contrast, even at a concentration 10-fold higher than the IC50 values for the various CPC 1:6 formulations, only a slight enhancement of antigen uptake was observed (1–1.5 relative antigen uptake) (Fig 5B and S3 Table). Thus, similar levels of uptake as the more toxic 6:1 CPC based NEs could be achieved with much reduced cytotoxicity using DODAC based NEs (Fig 5B and 5E). CTAC formulations also stood out in the screen as having less cytotoxicity than CPC/Tween80 1:6 with IC50 values near 0.45%, but intermediate levels of uptake (greater uptake than CPC/Tween80 1:6, but less than 6:1 CPC NEs and 1:6 and 6:1 DODAC NEs at the 0.05% concentration) (Figs 3 and 4). Cluster analysis again indicated that both cytotoxicity and antigen uptake activities tend to cluster according to the cationic surfactant identity and composition ratio (Fig 5). Only minor differences were seen between formulations with the same cationic and but different nonionic, and little clustering according to nonionic surfactant was observed for uptake and cytotoxicity (Fig 5C and S3 Fig). Thus, the NE library provides a set of compounds with a wide spectrum of activities: those with *high*, *intermediate or low* cytotoxicity together with *high*, *intermediate or low* NE-mediated cellular antigen uptake, allowing for an analysis of which activities and optimal surfactant combination yields adjuvant activity.

#### NF-κB Activation

A predominant pathway through which innate immunity is activated is by signaling through TLRs. The binding of pathogen-associated molecular patterns (PAMPs) to TLRs culminates in the activation of a common signaling pathway through the transcriptional activity of NFκB in antigen presenting cells [34, 35]. The ability of NEs to induce TLR activation was studied in Raw-Blue macrophages using an NFκB reporter assay for the select NEs used for the *in vivo* studies discussed below. These cells express mouse TLRs 1–9 (except TLR5) (as well as RIG-I, MDA-5, NOD1, NOD2, and Dectin-1), and stably express a gene for secreted embryonic alkaline phosphatase (SEAP) on a NFκB and AP-1 inducible promoter [10, 36].

**Fig 6:**
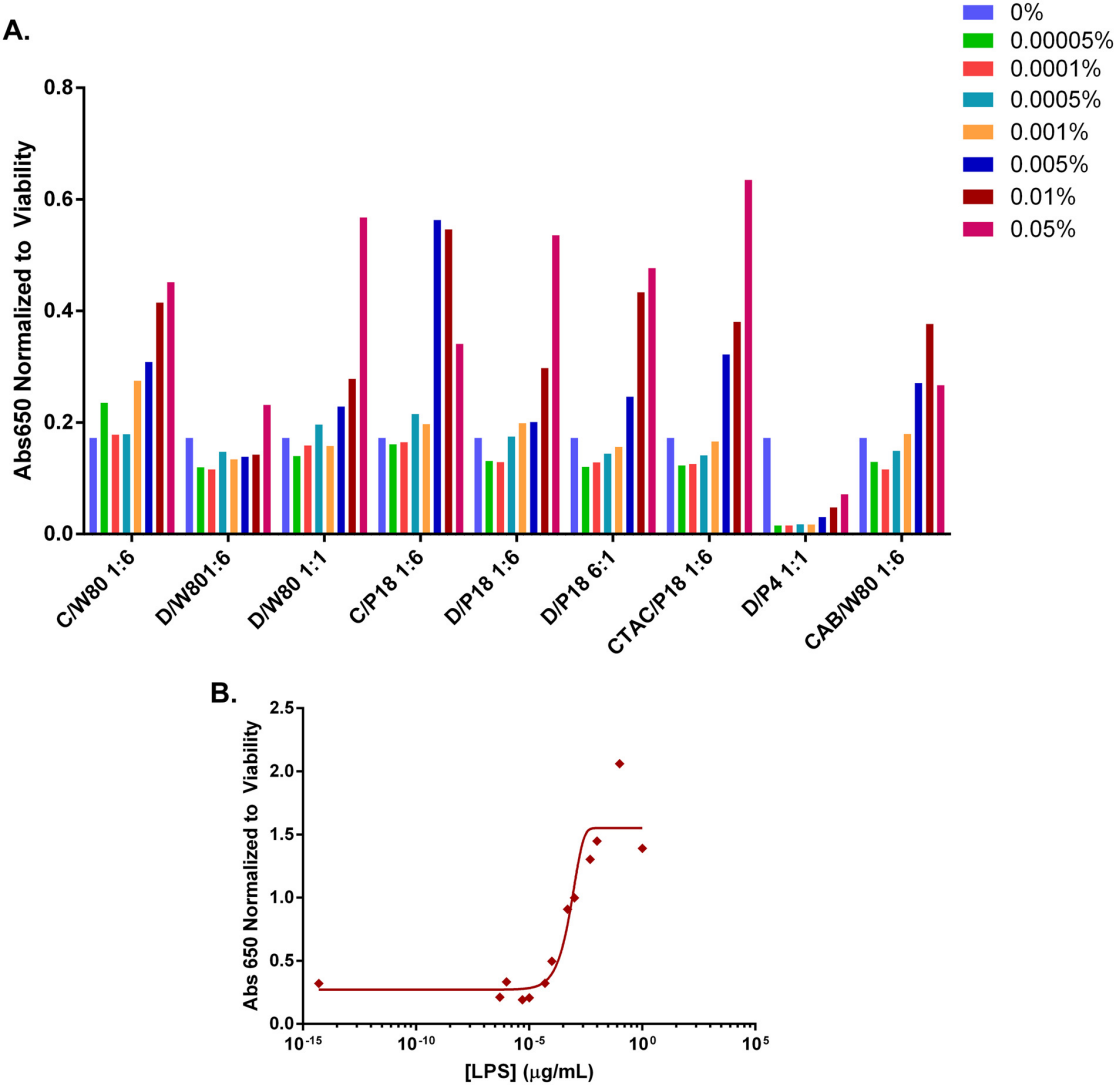
NF-κB activation for RawBlue macrophages. Cells were treated for 24 h at 37°C with increasing concentrations of (A) NE, or (B) LPS. The level of activation was detected by SEAP activity as measured by absorbance at 650 nm normalized to the relative number of viable cells remaining after the 24 h treatment assessed by XTT (S4 Fig). (Abbreviations CPC (C), Tween80 (W80), DODAC (D), P188 (P18), P407 (P4)).

RAW-Blue cells were treated with NE over a range of concentrations (5x10^-5^-1%) for 24 h, and TLR activation and subsequent NFκB transcriptional activation was measured by an increase in SEAP activity (Fig 6A). Treatment of RAW-Blue cells with the TLR4 agonist lipopolysaccharide (LPS), as a positive control resulted in significant induction of SEAP production with a logarithmic dose-response curve (Fig 6B). Several NEs were also found to induce TLR mediated NFκB activation in a dose-dependent manner. It is important to note that the NE concentration range shown in Fig 6A spans a range of concentrations below and above the IC50 values for several of the NEs. Further increasing the NE concentration beyond 0.1% resulted in a drop in SEAP activity for all NEs tested due to the effects of cytotoxicity. To account for the impact of *in vitro* cytotoxicity on SEAP production, SEAP activity was normalized to the number of viable cells remaining after 24h of NE treatment as measured by an XTT viability assay (S4 Fig).

DODAC/Tween80 1:6 and DODAC/P407 1:1 showed the least NF-κB activation at all concentrations tested. At the highest NE%, 0.05%, CTAC/P188 1:6, DODAC/Tween80 1:1, DODAC/P188 6:1, DODAC/P188 1:6, and CPC/Tween80 1:6 induced the highest levels of NF-κB activation. It is of note that while DODAC/Tween80 1:6 showed relatively no NF-κB activation, DODAC/P188 1:6 induced significant levels of SEAP production at both 0.01 and 0.05%, pointing to the role of the nonionic surfactant in influencing TLR activation. At lower NE concentrations such as 0.01% however, CPC/P188 1:6 induced the highest levels of NF-κB activation, followed by CPC/Tween80 1:6 and DODAC/P188 6:1and CTAC/P188 1:6. This demonstrates that less CPC is required to induce TLR activation than DODAC. However, the highest levels of TLR activation overall are seen for DODAC NEs with the highest DODAC ratio and NE%, likely due to the greater cytotoxicity of CPC. Interestingly CAB/Tween80 1:6 was also able to induce significant NF-κB activation, demonstrating that NE TLR activation is not dependent on the presence of a cationic charge.

Intriguingly, DODAC/P407 1:1 did not show activation of NF-κB through TLR activation, and in fact appeared to inhibit endogenous NF-κB activity. This NE did, however, induce immunogenicity *in vivo* as discussed below. Thus, one cannot rule out the possible involvement of TLR-independent pathways in the mechanism of NE immune activation.

### *In Vivo* Immunogenicity Studies

#### 3D Scatter Plot Correlations and Selection of NEs for In Vivo Studies

**Fig 7:**
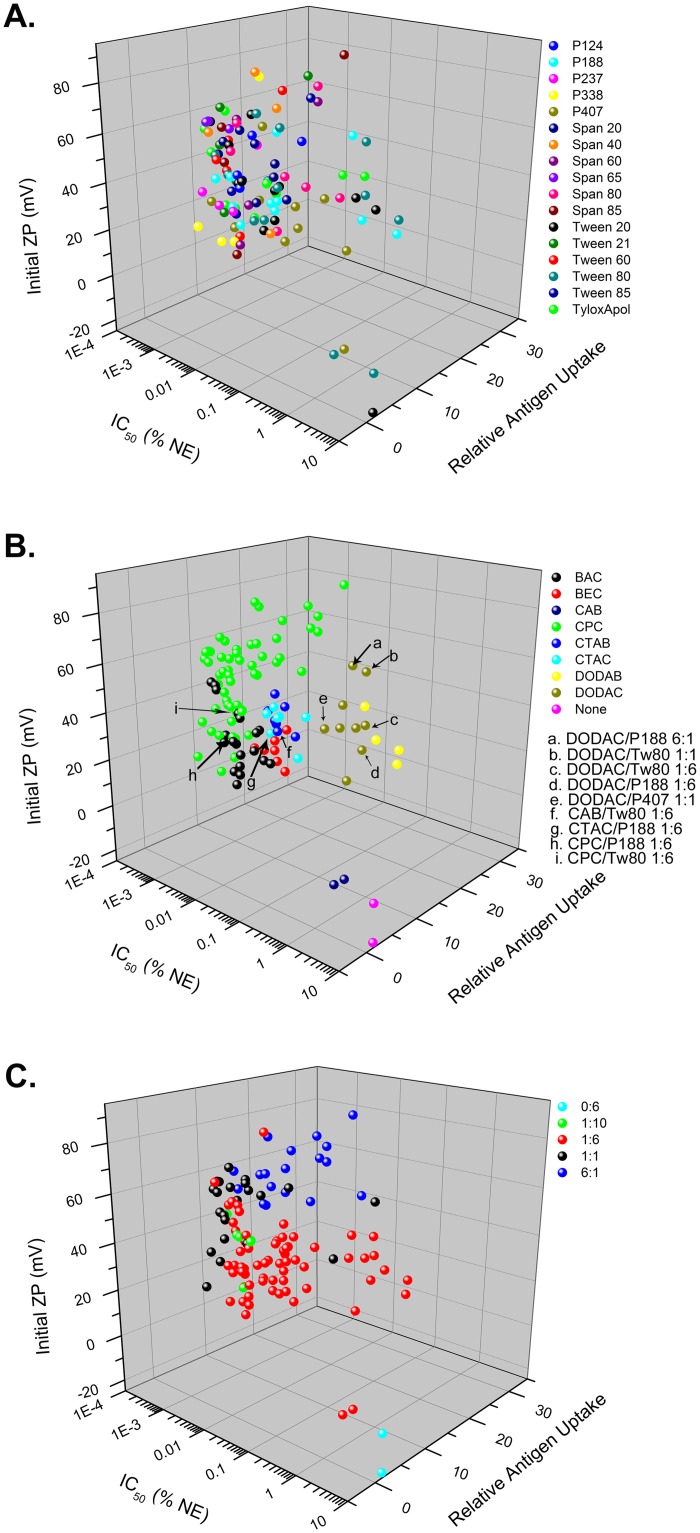
3D scatter plots of *in vitro* assay results for cytotoxicity (IC50), antigen uptake, and ZP_init_. Scatter plots are colored by (A) nonionic surfactant, (B) cationic surfactant, or (C) cationic:nonionic ratio. Formulations further evaluated *in vivo* are indicated by arrows.

The data from the aforementioned *in vitro* screening assays were plotted on 3D scatter plots to examine the relationship between the different biological activities, and to select candidate NEs for *in vivo* evaluation. Multi-parameter evaluation demonstrated correlations between certain biological and physico-chemical properties such as particle charge (ZP), cytotoxicity (IC50) and antigen uptake as dependent primarily on the cationic surfactant. Fig 7 shows cytotoxicity vs. relative antigen uptake vs. the ZP_init_ for all of the NEs. Fig 7A–7C show the same data but highlight different aspects of the NEs such as cationic or nonionic surfactant type and ratio. A clear clustering of NE activity by cationic surfactant type is seen in these plots as well as by cationic:nonionic surfactant ratio (Fig 7B and 7C). In contrast, Fig 7A shows the relationship between nonionic surfactants and the *in vitro* readouts which does not show clustering, suggesting that the nonionic surfactant does not play a defining role in dictating the *in vitro* parameters investigated. While several trends are apparent, such as greater charge being associated with mucoadhesion, and higher cytotoxicity and greater antigen uptake (with the exception of a few cationic surfactants), the complexity of the mechanism by which NEs activate immunogenicity is underscored by the lack of an obvious correlation between these parameters as well as with NF-κB activation.

**Fig 8:**
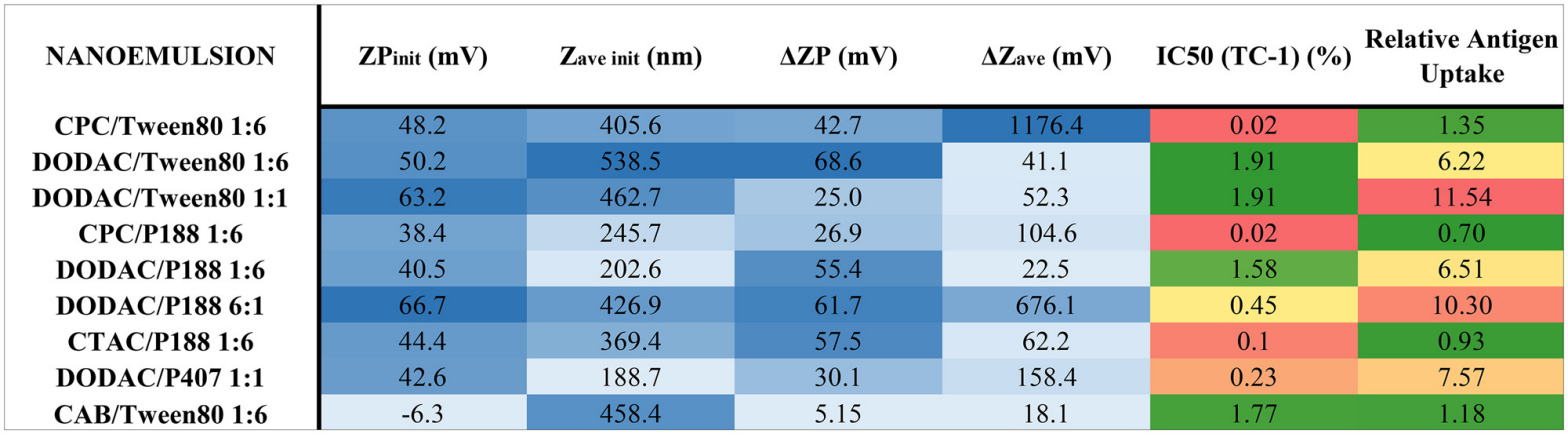
Summary of NE properties and results of *in vitro* screening assays for NEs selected for *in vivo* evaluation. Values are colored light to dark blue according to smallest to largest values of ZP_init_, Z_ave init_, ΔZP and ΔZave with mucin, and green to red for lowest to highest cytotoxicity and relative antigen uptake.

In order to further examine the influence of cationic and nonionic surfactants on immunogenicity, and to determine how the *in vitro* activities of the NE translate into biological outcomes *in vivo*, we selected a series of compounds from our screening assays that displayed distinct differences in their *in vitro* properties (ex. high uptake, low cytoxicity, mucoadhesive, high charge vs. high uptake, high toxicity, mucoadhesive, high charge, etc.) for further *in vivo* evaluation of adjuvant activity. NEs were chosen from different sectors of the 3D plot of IC50, relative antigen uptake, and ZP_init_ (Fig 7). Three different cationic surfactants CPC, CTAC and DODAC and the zwitterionic surfactant, CAB in combination with the nonionic surfactants, Tween80, P188 or P407 at various surfactant blend ratios were evaluated intranasally for adjuvant activity with the model antigen, OVA. The NEs chosen included CPC/Tween80 1:6, CPC/P188 1:6, DODAC/Tween80 1:6 and 1:1, DODAC/P188 1:6 and 6:1, DODAC/P407 1:1, CTAC/P188 1:6, and CAB/Tween80 1:6. The properties of these NEs are summarized below in Fig 8, and the locations of these NEs on the 3D scatter plots are indicated in Fig 7B. CPC NEs were selected as they showed the greatest cytotoxicity but relatively low levels of antigen uptake compared to DODAC NEs that had low cytotoxicity but very high levels of antigen uptake. DODAC NEs with higher cationic:nonionic ratios were also selected in order to evaluate the effects of increased cationic surface charge, high uptake and intermediate levels of cytotoxicity on immunogenicity *in vivo*. By comparing the same nonionic surfactants in combination with these cationic surfactants, the effects of variation of the nonionic component on immunogenicity was evaluated, as well as the effects of particle size, as P188 and P407 NEs are characterized by smaller Z_ave init_ values.

NEs with CPC ratios greater than 1:6 were not included as they were previously tested *in vivo*, and resulted in severe inflammation at the site of application, leading to poor tolerability (data not shown). The high inflammatory properties of these higher CPC NEs *in vivo* demonstrate the validity of the *in vitro* cytotoxicity screen in predicting irritation and toxicity *in vivo*. DODAC/Tween80 6:1, DODAC/P188 1:1, and DODAC/P407 1:6 and 6:1 were not included in any of our characterization as they did not form stable NEs.

#### Evaluation of Innate Cytokine Production Following Vaccination

**Fig 9:**
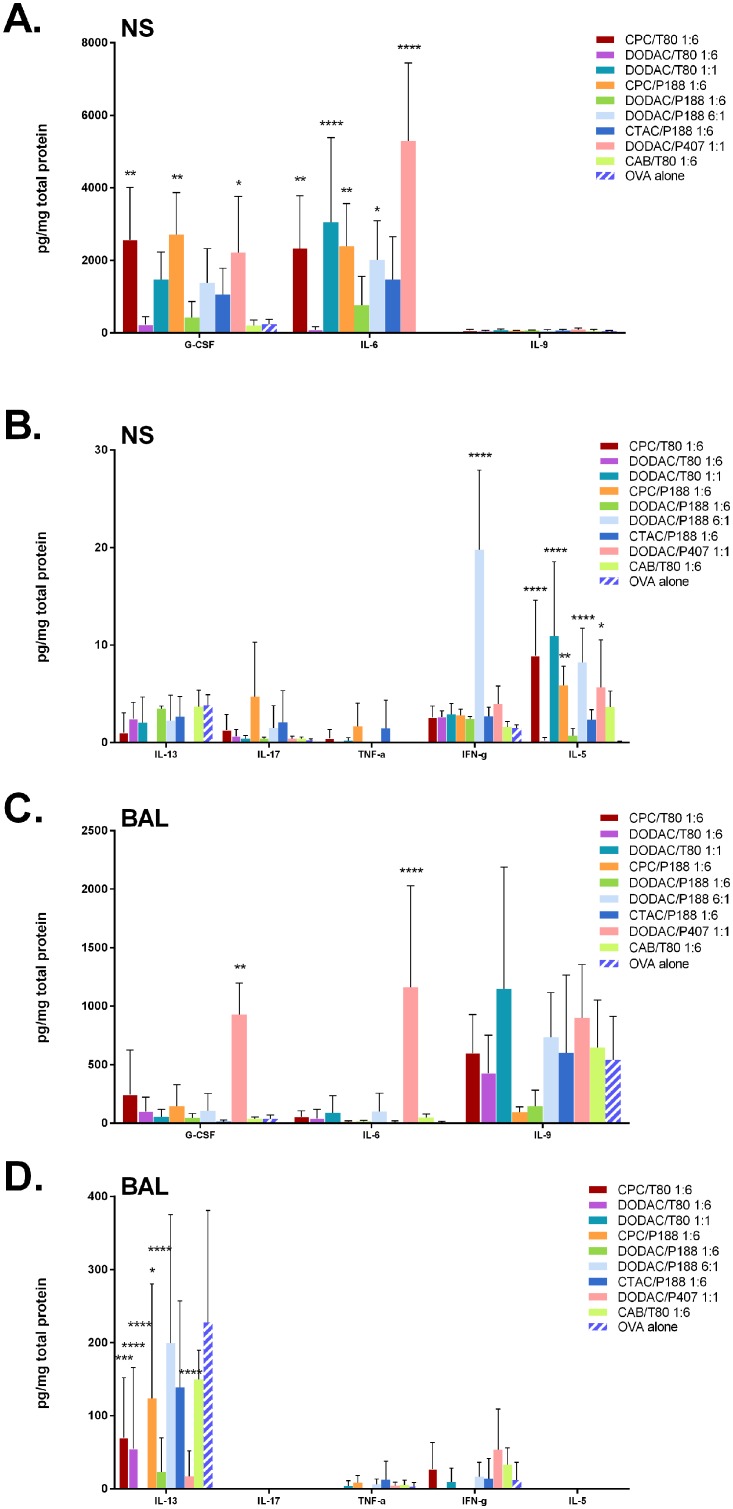
Acute phase cytokine profiles of C57/Bl6 mice. Cytokine levels measured 24 h after immunization with 20% NE with 20 mg/mL of OVA in 15 μL (7.5 μL/nare) (n = 4 per group). Levels of G-CSF, IFN-γ, IL-5, IL-6, IL-9, IL-13, IL-17, and TNF-α were measured by Milliplex and normalized to total protein concentration for **(A, B)** nasal septum homogenate (NS), and **(C, D)** bronchial alveolar lavages (BAL). (*p < 0.05, **p < 0.01, ***p < 0.001, ****p < 0.0001 as compared to OVA alone using 2-way ANOVA with Dunnett’s multiple comparison test).

To evaluate the acute cytokine response upon intranasal immunization, C57/Bl6 mice were immunized with the nine formulations of interest listed in Fig 8 (20% NE containing 20 μg OVA in 15 μL (7.5 μL per nare)). This dosage was determined to be optimal in previous studies [10, 19]. The animals were sacrificed after 24 h, after which the nasal septa were isolated and bronchial alveolar lavages (BAL) collected. Initial pilot studies were done in which the levels of 22 cytokines and chemokines were measured in the nasal septa and BAL during the acute phase for a few NE formulations. We selected 8 of the cytokines that showed evidence of local acute induction by NE treatment (G-CSF, IFN-γ, IL-5, IL-6, IL-9, IL-13, IL-17, and TNF-α) for further evaluation of the nine NEs of interest (Fig 9). Luminex analysis revealed distinct differences in the levels of cytokine/chemokine induction between the different NE formulations. CPC/Tween80 1:6 and CPC/P188 1:6 showed striking differences in their acute cytokine induction profiles as compared to their DODAC counterparts, DODAC/Tween80 1:6 and DODAC/P188 1:6 in the nasal septa even though the CPC and DODAC formulations have similar ZP and particle sizes as discussed above (Fig 9A and 9B). While immunization with CPC/Tween80 1:6 and CPC/P188 1:6 resulted in significant induction of G-CSF, IL-6, and IL-5 as compared to OVA alone, as well as some induction of TNF-α, DODAC/Tween80 1:6 and DODAC/P188 1:6 showed minimal, if any, increase in the levels of these cytokines relative to mice treated with OVA alone. Induction of these pro-inflammatory cytokines by the CPC formulations is consistent with the greater *in vitro* cytotoxic and inflammation/irritation inducing properties of CPC NEs compared to DODAC NEs.

Besides IL-5, the levels of all the cytokines studied induced by the zwitterionic CAB/Tween80 1:6 were very low and were equivalent to the levels induced by OVA alone, highlighting the importance of cationic charge. However, cytokine levels induced by DODAC/Tween80 1:6 were lower than or equivalent to those elicited by CAB/Tween80, demonstrating that cationic charge is necessary, but not sufficient for inducing an acute response.

*In vivo* studies of CPC NEs were limited to the 1:6 ratio, as NEs with higher CPC:nonionic ratios such as 1:1 and 6:1 had previously resulted in excessive inflammation. In contrast, DODAC NEs were well tolerated *in vivo* even at ratios of 6:1. Increasing the DODAC/Tween80 ratio from 1:6 to 1:1 markedly increased the average levels of G-CSF (6-fold), IL-6 (35-fold) and IL-5 (61-fold) relative to DODAC/Tween80 1:6 (Fig 9A and 9B). At the 1:1 ratio, DODAC/Tween80 treated mice in fact, had average IL-6 and IL-5 values greater than CPC/Tween80 1:6, and similar levels of TNF-α in the nasal septa as the CPC NE (Fig 9A and 9B). DODAC/P407 1:1 elicited the highest levels of IL-6 production in the nasal septa compared to all NEs tested, and had similar levels of G-CSF, IL-5, and TNF-α as CPC/Tween80 and CPC/P188 1:6. The IC50 values of DODAC/Tween80 and DODAC/P407 1:1 were 0.8377% and 0.2280% in TC-1 cells which were lower than that of DODAC/Tween80 1:6, but still higher than that of CPC/Tween80 1:6 of 0.0170%. These results show the relationship between cytotoxicity observed *in vitro*, and production of pro-inflammatory cytokines *in vivo*. Furthermore, the dramatic differences between the cytokine profiles of CPC and DODAC NEs demonstrate that the dominant influence of the cationic surfactant type and ratio on NE activity observed *in vitro* applies to NE activity *in vivo* as well.

Interestingly, even at the highest ratio of 6:1, DODAC/P188 elicited lower or equivalent levels of G-CSF, IL-6, IL-17, and TNF-α and only a minimal enhancement of IL-5 production as compared to CPC/P188 1:6. At this high cationic concentration, however, DODAC/P188 6:1 induced very high amounts of IFN-γ >7-fold higher than the other formulations tested. It was the only formulation that significantly enhanced IFN-γ relative to OVA alone. Lastly, CTAC/P188 1:6, which gave cytotoxicity (IC50 of 2.937% in TC-1 cells) greater than the DODAC 1:6 formulations and lower than all CPC formulations elicited an intermediate acute response with levels of G-CSF, IL-6, TNF-α, and IL-5 greater than the 1:6 DODAC NEs, but less than the CPC NEs. This finding again appeared to support a relationship between cytotoxicity observed *in vitro* and immunogenicity.

A different pattern of cytokine expression was observed in the BAL. In BAL, NE mediated cytokine production was similar to that observed with antigen alone (Fig 9C and 9D). Only DODAC/P407 1:1 induced higher levels of IL-6 and significantly higher levels of G-CSF than OVA alone or with other NEs. Notably, this NE also elicited the highest levels of IL-6 in the nasal septum. Interestingly, DODAC/P188 6:1 which gave very high IFN-γ in the nasal septum did not induce IFN-γ production in the BAL. IL-17, TNF-α, and IL-13 levels did not appear to be above those seen with OVA alone for any of the NEs in either the nasal septum or BAL. Thus, it is clear that different surfactant combinations give different acute cytokine profiles.

#### Evaluation of Cellular and Humoral Adaptive Immune Responses

**Fig 10:**
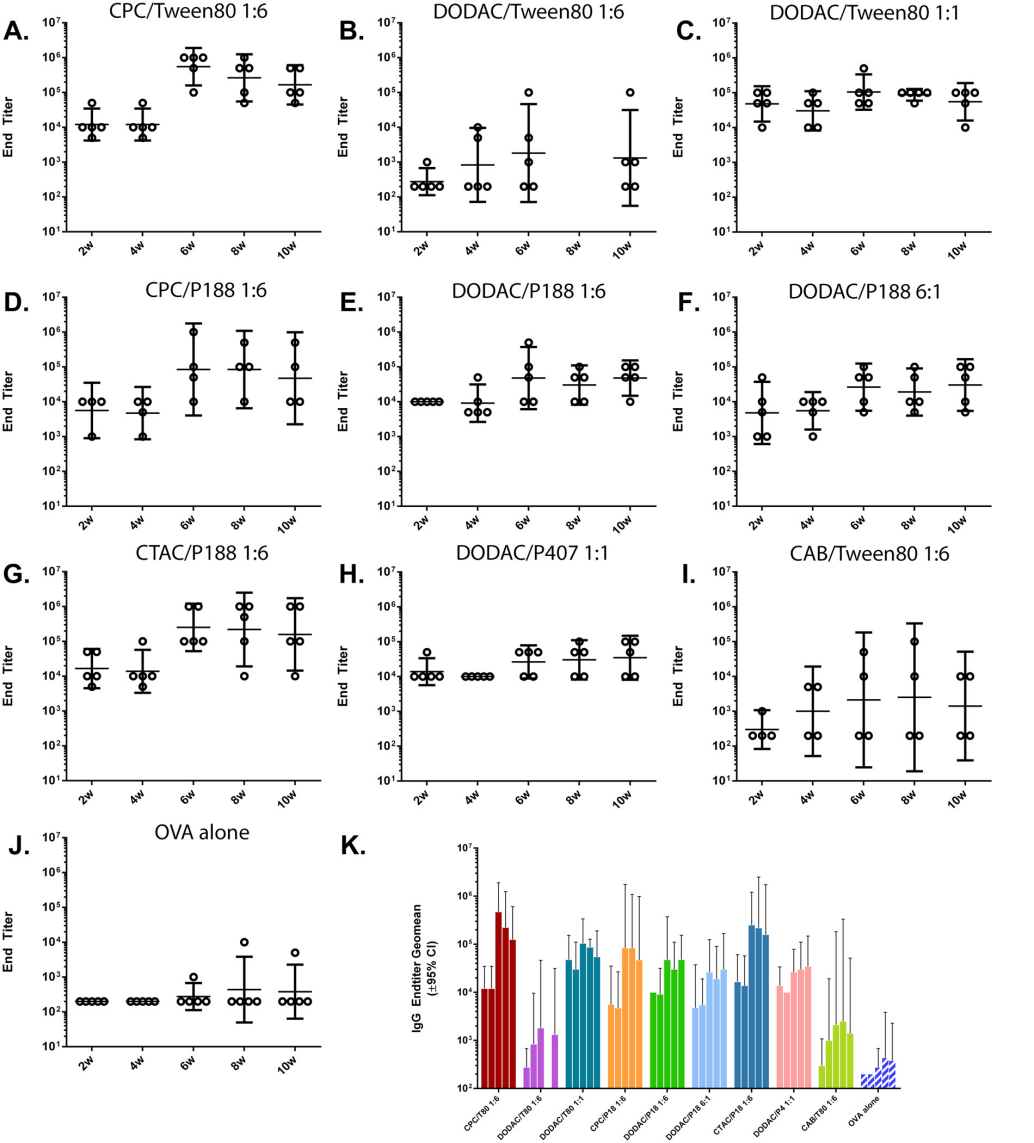
Serum OVA-specific IgG titers. IgG was measured after IN immunization with 20% NE with 20 μg of OVA or OVA alone. Immunizations were performed at 0 and 4 weeks (after the 4 week serum collection). **(A)** CPC/Tween80 1:6, **(B)** DODAC/Tween80 1:6, **(C)** DODAC/Tween80 1:1, **(D)** CPC/P188 1:6, **(E)** DODAC/P188 1:6, **(F)** DODAC/P188 6:1, **(G)** CTAC/P188 1:6, **(H)** DODAC/P407 1:1, **(I)** CAB/Tween80 1:6, **(J)** OVA alone. Geometric means are indicated±95% Cl. **(K)** Endtiter geometric means plotted ±95% Cl.

To further examine the relationship between NE *in vitro* activity and immunogenicity, both humoral and cellular adaptive immune responses were evaluated for the nine NEs. C57BL/6 mice (n = 5 per group (n = 4 for CPC/P188 1:6 and CAB/Tween80 1:6)) were intranasally immunized twice, 4 weeks apart, with a mixture of 20% NE and 20 μg OVA, or OVA alone in PBS as a control. Serum anti-OVA specific IgG was evaluated at 2, 4, 6, 8 and 10 weeks post-initial immunization. This dosage and treatment duration has been previously determined as optimal (data not shown) [19]. While immunization with any OVA/NE combination resulted in higher IgG endpoint titers compared to OVA alone, notable differences in serum IgG kinetics and end-point titers were observed, which were dependent on the NE surfactant composition. (Fig 10). Since an optimal antigen concentration was used in these studies, a stronger adjuvant effect was best delineated by a greater number of high antibody responders per group, yielding a smaller spread in the range of IgG titers (Fig 10). These differences between the NEs are expected to be magnified at suboptimal antigen dosages.

Zwitterionic CAB/Tween80 1:6 and DODAC/Tween80 1:6 showed no significant increase in OVA-specific IgG compared to OVA alone after 2 weeks. With the exception of DODAC/Tween80 1:6, however, all NEs with cationic surfactant showed a robust OVA-specific serum IgG response at 2 weeks. Four NE formulations stimulated early IgG responses with equivalent (DODAC/P188 1:6, DODAC/P407 1:1, CTAC/P188 1:6) or higher (DODAC/W80 1:1) (>0.5 logs) end-titers as compared to CPC/Tween80 1:6, 2 weeks following intranasal priming with NE-OVA (Fig 10K). Two additional formulations (CPC/P188 1:6 and DODAC/P188 6:1) had slightly lower average IgG responses initially at weeks 2–4, but this was attributable to the larger spread in the IgG response due to the presence of 1–2 low responders (Fig 10D and 10F).

Again with the exception of CAB/Tween80 1:6 and DODAC/Tween80 1:6, IgG increased dramatically after the booster immunization at 4 weeks for the 7 NEs. There was a discernible difference in the responsiveness to the boost immunization for the different NEs. While the pre-boost IgG titer of DODAC/Tween80 1:1 was >0.5 log higher than that of CPC/Tween80 1:6, upon boost, the titer of CPC/Tween80 1:6 increased by >1.5 logs as compared to only a ~0.5 log increase for DODAC/Tween80 1:1 (Fig 10A and 10C). This resulted in higher IgG titers for CPC/Tween80 1:6 after the boost compared to DODAC/Tween80 1:1 (Fig 10K). A similar pattern was observed for DODAC/P188 1:6 and CPC/P188 1:6 where DODAC/P188 1:6 had equivalent or slightly higher pre-boost titers, but lower post-boost titers than CPC/P188 1:6 (increased by ~0.5 log vs. 1.25 logs post-boost, respectively) (Fig 10D and 10E). Even with higher concentrations of DODAC, DODAC/Tween80 1:1, DODAC/P188 6:1 and DODAC/P407 1:1 only showed a ≤0.5 log increase in IgG after the boost, never achieving the high titers observed for the CPC formulations (> 1 log higher) with the same nonionic surfactant at 10 weeks. CTAC/P188 1:6, however showed a similar increase of 1 log in IgG post-boost and gave final titers similar to the CPC NEs. These results suggest that DODAC formulations do not boost as sensitively and are not as immunogenic as CPC and CTAC NEs. These results are in agreement with the findings of the acute response studies demonstrating the strong relationship between greater *in vitro* cytotoxicity/inflammation and greater immunogenicity for these NEs.

Of interest, is the order of magnitude higher IgG titers of DODAC/Tween80 1:1 relative to CPC/Tween80 1:6 pre-boost at 2–4 weeks. More studies are necessary; however, this could suggest faster kinetics for DODAC/Tween80 1:1, making it a candidate for applications in which a single immunization schedule is desirable.

While there was minimal IgG production after 2 weeks for DODAC/Tween80 1:6 and CAB/Tween80 1:6, there was still a minor adjuvant effect, as after 4 weeks, IgG increased by 1.5–2 logs for some of the mice in these treatment groups. CAB/Tween80 1:6 induced a poor IgG response detectable in only 2 of 4 mice in this group, with the other 2 showed no response even after the second immunization (Fig 10I). In previous *in vivo* studies we have shown that Tween 80 0:6 containing no cationic surfactant also elicits a similar minimal IgG response [19]. These results again demonstrate the importance of NE cationic charge for immunogenicity. However, even with the same magnitude of cationic charge as CPC/Tween80 1:6, DODAC/Tween 80 1:6 induced a poor IgG response, giving a similar spread as the CAB/Tween80 1:6 group. 2 out of 5 mice showed no response at all, and others had only low IgG titers ranging from 1–3 orders of magnitude lower than CPC/Tween80 1:6. These results were consistent with acute phase cytokine profiles in that neither NE enhanced cytokine levels in the nasal septum or BAL, and indicate that a positive charge is necessary but not sufficient to induce immunogenicity.

OVA-specific IgA responses in bronchial lavage from the immunized animals were low for all formulations tested in this study (S5 Fig). The low IgA may be antigen specific, as we have previously demonstrated induction of significant levels of antigen-specific mucosal IgA towards other recombinant proteins and whole viral particles with CPC/Tween80 1:6 [9, 11, 13].

**Fig 11:**
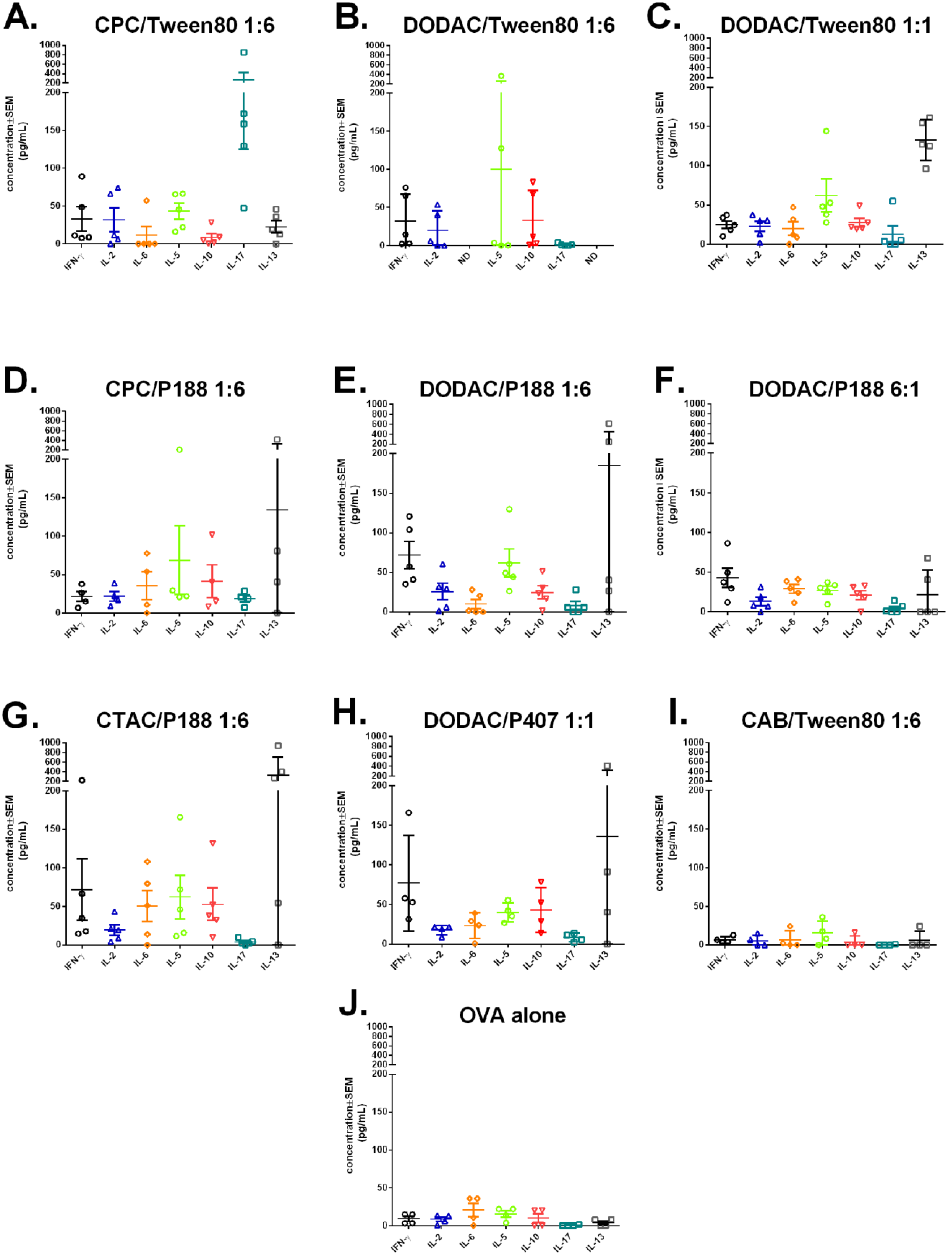
Cell-mediated immune response in splenocytes. Cytokine profiles of restimulated splenocytes isolated from mice after two immunizations at 10 weeks post initial immunization with (A) CPC/Tween80 1:6, (B) DODAC/Tween80 1:6, (C) DODAC/Tween80 1:1, (D) CPC/P188 1:6, (E) DODAC/P188 1:6, (F) DODAC/P188 6:1, (G) CTAC/P188 1:6, (H) DODAC/P407 1:1, (I) CAB/Tween80 1:6, or (J) OVA alone. Means are plotted ± SEM (ND = not determined).

Cell-mediated immune responses were evaluated in splenocytes isolated at sacrifice on week 10. Splenocytes were stimulated with full-length OVA to evaluate the profile of cytokines secreted by CD4+ T-cells (Fig 11). OVA stimulation yielded different cellular response profiles (T_H_1, T_H_2, T_H_17) in animals immunized with NEs with different surfactant compositions. As expected, minimal cytokine production was induced for CAB/Tween80 1:6 immunized mice, which gave levels equivalent to mice immunized with OVA alone. Interestingly, while the OVA alone treated mice produced minimal anti-OVA IgG, some of the CAB/Tween80 1:6 immunized mice produced IgG titers comparable to some of the high cytokine producing NEs, such as DODAC/Tween80 1:1. The other NEs tested gave mixed responses with both T_H_1 and T_H_2 cytokines produced at different ratios depending on the formulation. While elevated IL-5 and IL-13 production was observed for several of the NEs, (particularly DODAC/Tween80 1:6, DODAC/Tween80 1:1, CPC/P188 1:6, DODAC/P188 1:6, CTAC/P188 1:6 and DODAC/P407 1:1), several of these NEs also showed the strongest induction of the T_H_1-type cytokines, IFN-γ and IL-2 (DODAC/Tween80 1:6, DODAC/P188 1:6, CTAC/P188 1:6, DODAC/P407 1:1). Of note, simply increasing the cationic:nonionic ratio did not result in an increase in cytokine production, as DODAC/P188 6:1 gave reduced or equivalent levels of cytokines compared to DODAC/P188 1:6. This pattern was also reflected in the IgG titers for the 6:1 NE. CPC/Tween80 1:6 which gave the highest levels of IgG was characterized by a more mixed T_H_1/T_H_2 response, slightly more skewed towards T_H_1. Stimulated splenocytes from this group produced intermediate levels of IFN-γ and IL-2, as well as intermediate levels of IL-5. Additionally, CPC/Tween80 1:6 was one of the lowest inducers of IL-13, and IL-10. None of the NEs tested induced detectable levels of IL-4, further supporting a slightly more T_H_1 response (S6 Fig). In addition, our prior studies have documented that immunization with CPC/Tween80 1:6 with several different antigen types induces both CD4 and CD8 cells that produce IFN-γ, and antibody isotypes compatible with a T_H_1 response (greater prevalence of IgG2b and IgG2a over IgG1) [8, 11, 15]. Together, these data suggest a T_H_1 skewing of the immune response. Most striking, however, was the marked reduction of the T_H_17 response when CPC was replaced with DODAC or CTAC. CPC/Tween80 1:6 induced very high levels of IL-17 (200–1000 pg/mL), however, DODAC/Tween80 1:6 induced no detectable IL-17. (CPC/Tween80 1:6 vs. DODAC/Tween80 1:6 p < 0.05, CPC/Tween80 1:6 vs. CAB/Tween80 1:6 or OVA alone p < 0.01 by Kruskal-Wallis with Dunn’s multiple comparisons test). Even increasing the DODAC to 1:1 and 6:1 ratios did not yield significant levels of IL-17. A different cationic surfactant, CTAC (CTAC/P188 1:6) was also unable to elicit IL-17 (Fig 11A, 11B, 11C, 11F and 11G). This occurred despite maintenance of the T_H_1 responses with the DODAC and CTAC formulation, and was not related to either the overall charge or cationic surfactant concentration (Fig 11). This dependence of the Th17 response on the identity of the cationic surfactant was also reflected by CPC/P188 1:6 which, while eliciting less IL-17 than CPC/Tween80 1:6, produced more than DODAC/P188 1:6 and DODAC/P188 6:1 which produced no IL-17 (Fig 11C, 11D and 11E).

There was also divergence in cellular and humoral responses with some formulations such as DODAC/Tween 80 1:6 which yielded suboptimal induction of serum antibodies but demonstrated cell mediated responses skewed towards substantial production of IFN-γ and IL-5 without IL-17 (Fig 11B). Collectively, these data suggest that both the magnitude and the type of immune response elicited by the NEs can be modulated for diverse applications by the interchange of different cationic or nonionic surfactants and by optimizing their surfactant ratios.

## Discussion

Mucosal vaccination can elicit cellular immunity as well as both mucosal and systemic antibody responses making it highly desirable for prophylactic vaccines against infections with mucosal pathogens. In these studies we formulated a series of stable nanoemulsions for use as mucosal adjuvants using combinations of nonionic surfactants, cationic surfactants, co-solvents and soybean oil. These formulations were evolved from an initial NE adjuvant, which contains CPC and Tween80 in a 1:6 ratio and has demonstrated protective mucosal immunity against multiple pathogens *in vivo* [7–9, 11–17]. We systematically varied the surfactant composition in order to further understand the mechanism of NE mediated adjuvant activity and determine the critical factors that dictate the type of immune response elicited *in vivo*. Varying the surfactant composition resulted in NEs of various particle sizes and surface charge—physical characteristics that we hypothesized play a role in adjuvant activity. Using high-throughput screening assays we assessed NE mucoadhesion, cellular uptake, and cytotoxicity, and found that these activities were dependent primarily on the cationic surfactant and the cationic:nonionic surfactant ratio of each formulation. Scatter analyses of these *in vitro* results revealed clustering of activity according to cationic surfactant type and ratio, with lesser influences from the nonionic surfactant component. Cationic surfactant type and ratio were also important to the induction of NF-κB activity *in vitro*, another activity thought to enhance adjuvanticity. While the importance of cationic charge in inducing immunogenicity has been demonstrated in other adjuvant systems including liposomes and polymer based nanoparticles, NE surface charge alone did not determine adjuvanticity [23–25, 28, 37–41]. Several NEs with identical surface charge showed strikingly different activities *in vitro* as well as *in vivo*. Thus, additional physicochemical properties of the cationic surfactant, such as hydrophobicity, head group geometry and tail structure and length must play important roles in defining NE adjuvant activity.

Multiple potential relationships could be inferred between different *in vitro* activities of NEs and their *in vivo* cellular and humoral immune response profiles. NEs provide an adjuvant effect in part by increasing the cellular antigen uptake through either a depot effect or by facilitating uptake into epithelial cells and dendritic cells. As we have previously demonstrated that adhesion to mucin *in vitro* correlates with increased retention of NE-antigen at the nasal mucosa and increased cellular uptake *in vivo* [19],we screened each formulation for the ability to bind mucin. Cationic surface charge on the NE was necessary for mucous adhesion and increasing the NE surface charge for each surfactant pair increased mucin association. However, while NEs with similar degrees of positive charge but different cationic surfactant types all bound to mucin, the ΔZ_ave_ values of this interaction were very different. For example, CPC/Tween80 1:6 and DODAC/Tween80 1:6, have similar surface charge and both bound mucin; however, CPC/Tween80, showed a much larger increase in particle size in the presence of mucin. While the implications of these differences are unclear, the findings suggest that the NEs may have distinct types of interaction with the mucosal layer upon intranasal application, and this is dependent on the surfactant composition.

Previously we have shown that the CPC/Tween80 1:6 NE formulation enhances antigen uptake in dendritic cells through the phagocytosis of apoptotic epithelial cells containing NE-antigen, and that this process is important to adjuvant activity as seen for other emulsion based adjuvants as well [18–20, 27]. Cluster analysis revealed that in general, NEs with the greatest positive charge showed the highest levels of antigen uptake, and increasing the cationic:nonionic ratio for each particular cationic/nonionic surfactant pair dramatically enhanced uptake. This is not surprising, as cationic charge in proximity to the cell surface is likely to be recognized as danger signal, stimulating phagocytosis of the NE-Ag complex [42–44]. Furthermore, the cationic surfactants relative rank order of antigen uptake was maintained even when these surfactants were paired with diverse nonionic surfactants. However, other factors appeared important in this process as well; for example, DODAC NEs were able to induce the highest levels of cellular uptake with the lowest amount of cationic surfactant compared to the other cationic surfactants. DODAC is the only cationic surfactant with two acyl chains which may allow these NEs to more effectively fuse with the cellular membrane, facilitating cellular delivery of protein antigens [30, 38, 45–47]. In contrast, at higher cationic ratios (ex. 6:1) CPC NEs gave the highest level of uptake of all the NE formulations—more than twice that of the 6:1 DODAC NEs. Confocal microscopy also showed striking differences in the antigen localization in cells exposed to CPC/Tween80 1:6 and 6:1, and DODAC/Tween80 1:6. OVA appeared to be localized in endosomes or lysosomes for CPC/Tween80, but was more uniformly distributed throughout the cytoplasm with DODAC/Tween80. This difference in antigen uptake was also associated with divergent immunogenicity *in vivo*, as CPC/Tween80 1:6, which had lower levels of uptake than DODAC/Tween80 1:6 induced more potent antibody and cellular responses *in vivo* than DODAC/Tween80 1:6. Thus, while the adjuvant activity of NEs involves cellular antigen uptake it also relates to complex factors of antigen localization and processing/presentation.

Another factor we felt was important to examine in evaluating adjuvanticity was immunogenic cell death [32]. During immunogenic apoptosis, secretion of damage-associated molecular patterns (DAMPs) by the dying cell can activate a specific T cell response through various routes of dendritic cell activation [22, 32, 48]. We evaluated this *in vitro* by measuring the cytotoxicity of the NEs towards three different cell types (epithelial, dendritic, and macrophage cells). The trends in relative cytotoxicity were maintained for all these cell types. In general, higher charge and cationic surfactant ratios for each cationic-nonionic surfactant pair increased the cytotoxicity, and there was a strong positive correlation between cytotoxicity and antigen uptake. DODAC NEs however, were exceptions to the trend, showing remarkably low cytotoxicity compared to other cationic NEs with the same charge, yet facilitating much higher levels of uptake. A correlation between toxicity and uptake is not surprising, since cells with higher intracellular levels of surfactant are likely less viable as cationic surfactants are disruptive to cellular membranes [49–51]. Furthermore, cationic surfactants have been shown to destabilize membrane lipid microdomains, and could change other membrane features, such as the curvature [44]. Furthermore, danger signals possibly presented by the NE likely stimulate pro-apoptotic and pro-inflammatory pathways classically activated by cationic molecules (ex. lysine, spermine) or cationic liposomes and drug compounds [31, 52–54].

Variation of the surfactant compositions of the formulations yielded NEs with different degrees of charge, mucoadhesion, antigen uptake, and cytotoxicity, which varied greatly in their ability to activate NF-κB *in vitro*, and in their stimulation of adjuvant immune responses *in vivo* after intranasal immunization. Small changes in surfactant composition could alter or even abrogate the adjuvant activity. None of this activity involved a biological ligand or macromolecule, but the surfactants used in the NEs have chemical structures which somewhat resemble TLR agonists and demonstrated TLR mediated NF-κB activation *in vitro*. DODAC/Tween80 1:6 induced the least NF-κB activation *in vitro*, and acutely elicited the lowest levels of NF-κB regulated cytokines in the nasal septum *in vivo*. In contrast, CPC NEs at all ratios induced greater NF-κB activation than DODAC/Tween80 1:6 *in vitro* and also induced higher levels of NF-κB regulated pro-inflammatory cytokines *in vivo*. Increasing the cationic ratio in DODAC NEs increased NF-κB activation *in vitro*, and accordingly increased the acute production of cytokines *in vivo*. The basis for these differences is unclear, but may be related to the charge being localized on a pyridinium ring on the CPC head which has a planar shape, allowing it to pack more efficiently at the NE oil-water interface, resulting in a higher charge density on the NE surface than the other cationic detergents in which the quaternary ammonium is localized to an akylammonium group which may have less efficient packing and lower charge density. Intriguingly, DODAC/P407 did not activate TLR and NF-κB and actually inhibited endogenous NF-κB activity *in vitro*. Despite this it induced cytokines *in vivo* and gave robust IgG titers, suggesting TLR-independent immune activation potentially through molecules such as TRIF. This may parallel what has been reported with cationic lipids which both can activate TLRs [11, 15, 44] and TRIF [3, 55, 56].

From the observed humoral and cellular recall responses it appeared we could tailor the immune response by manipulating the *in vitro* cytotoxic activity by altering the cationic surfactant type, cationic:nonionic ratio and the nonionic surfactant type. Serum antibody responses 10 weeks after immunization were related to the degree of cytotoxicity observed *in vitro* and were associated with acute production of the pro-inflammatory cytokines G-CSF and IL-6. (Fig 10K). Minimally cytotoxic NEs that did not induce acute cytokine production also failed to induce a robust humoral IgG response. A more divergent cellular response (T_H_1, T_H_2, T_H_17) was seen even with different formulations that had similar sizes, charges and serum IgG responses. All of the NEs tested (except CAB/Tween80 1:6) elicited a mixed T_H_1 and T_H_2 response, eliciting both T_H_1 and T_H_2 cytokines. Variation of the surfactant composition type and ratio, however, altered the relative levels of these T_H_1 and T_H_2 cytokines even for NEs with the same charge and particle size. The most dramatic difference was the absence of a T_H_17 response when CPC was replaced with DODAC or CTAC, for NEs with either Tween80 or P188 nonionic surfactants. Furthermore, even in the absence of a robust serum IgG response, DODAC/Tween80 1:6 showed notable production of IFN-γ and IL-2 as well as IL-5 and IL-10 without induction of IL-17 (Fig 11B). The reason for the selective activation of T_H_17 by some emulsions in not clear, but has only been observed when these formulations are used on mucosal surfaces, rather than administered intramuscularly or subcutaneously (unpublished data). In addition, cellular uptake of antigen followed by epithelial cell apoptosis appeared central to this process. This may suggest that the unique formulations that induce IL17 production are replicating the events that occur during a respiratory viral infection: cellular infection and TLR activation, followed by apoptosis and dendritic cells sampling. Only when all these events occur concurrently does T_H_17 get activated, even though all three routes of administration can elicit similar humoral responses. It may be that this degree of regulation is important since T_H_17 immunity is crucial in local mucosal protection but clearly detrimental in systemic inflammatory diseases.

In summary, we have shown that by varying the surfactants and the surfactant blend ratio used to formulate a nanoemulsion adjuvant, we could modulate the magnitude and kinetics of the antibody response as well as skew the cellular response (T_H_1, T_H_2 and T_H_17). These findings have important implications on the use of nanoemulsion adjuvants because they suggest that the adjuvants can be chosen based on the type of immune response necessary to protect from a particular pathogen. While challenge studies with additional antigen types will provide important information regarding the implications of these findings, collectively, these results demonstrate the value of using high-throughput screens to aid in the design of custom adjuvants.

## Supporting Information

S1 Fig Summary of mucoadhesion properties for all NE formulations. **(A)** Change in particle size and **(B)** ZP from Figs 1 and 2D scatter analysis of mucoadhesion properties for all NE formulations colored by cationic surfactant plotted by **(C)** ΔZ_ave_ vs. ΔZP, **(D)** ΔZP vs. ZP_init_, **(E)** ΔZ_ave_ vs. ZP_init_.
(TIF)

S2 Fig Viability curves for select NEs in Jaws II cells. Curves shown are the average of two independent experiments ± SD.
(TIF)

S3 Fig Scatter plots of relative antigen uptake vs. IC50. Scatter plots are shown for **(A)** 1:1 and **(B)** 6:1 NEs. **(C)** Scatter plots of antigen uptake vs. IC50 for all NEs tested at all ratios (same data as Fig 4A) colored by DODAC NEs (blue) and non-DODAC NEs (orange).
(TIF)

S4 Fig Relative viability of Raw-Blue cells. Relative viability of Raw-Blue cells used for NFκB activation studies as measured by an XTT assay after (A) 24h NE treatment or (B) LPS treatment from which supernatants were assessed for SEAP activity. Final treatment concentrations are expressed as % for NE and μg/mL for LPS.
(TIF)

S5 Fig IgA responses in BAL. OVA-specific IgA from immunized mice measured in BAL is shown as OD at 405 nm ± standard deviation.
(TIF)

S6 Fig IL-4 levels from restimulated splenocytes isolated from mice after two immunizations at 10 weeks post initial immunization. Groups include OVA alone, CPC/Tween80 1:6, DODAC/Tween80 1:6, DODAC/Tween80 1:1, CPC/P188 1:6, DODAC/P188 1:6, DODAC/P188 6:1, CTAC/P188 1:6, DODAC/P407 1:1, or CAB/Tween80 1:6 (same samples used for analysis in Fig 9). Means are plotted ± SEM. Samples were analyzed using a Milliplex MAP polystyrene multiplex kit. (Abbreviations Tween80 (T80), DODAC (D), P188 (P18), P407 (P4)).
(TIF)

S1 Table Complete summary of *in vitro* assay results for all NE formulations. **(A)** particle size (initial diameter) of NE alone, **(B)** ΔZ_ave_ with mucin. (Z_ave_, ΔZ_ave_, ZP, ΔZP, antigen uptake, cytotoxicity).
(TIF)

S2 Table Complete summary of *in vitro* assay results for all NE formulations. **(A)** ZP_init_, and **(B)** ΔZP with mucin.
(TIF)

S3 Table Complete summary of *in vitro* assay results for all NE formulations. relative antigen uptake in TC-1 cells.
(TIF)

S4 Table Complete summary of *in vitro* assay results for all NE formulations. cytotoxicity results expressed as IC50 values in (A) TC-1, (B) JAWS II, and (C) RAW cells.
(TIF)
