# Supplementary figures and images for: Formulation, High Throughput In Vitro Screening and In Vivo Functional Characterization of Nanoemulsion-Based Intranasal Vaccine Adjuvants
Source: PLoS One. 2015 May 11;10(5):e0126120. doi: 10.1371/journal.pone.0126120 (PMC4427474; doi:10.1371/journal.pone.0126120)

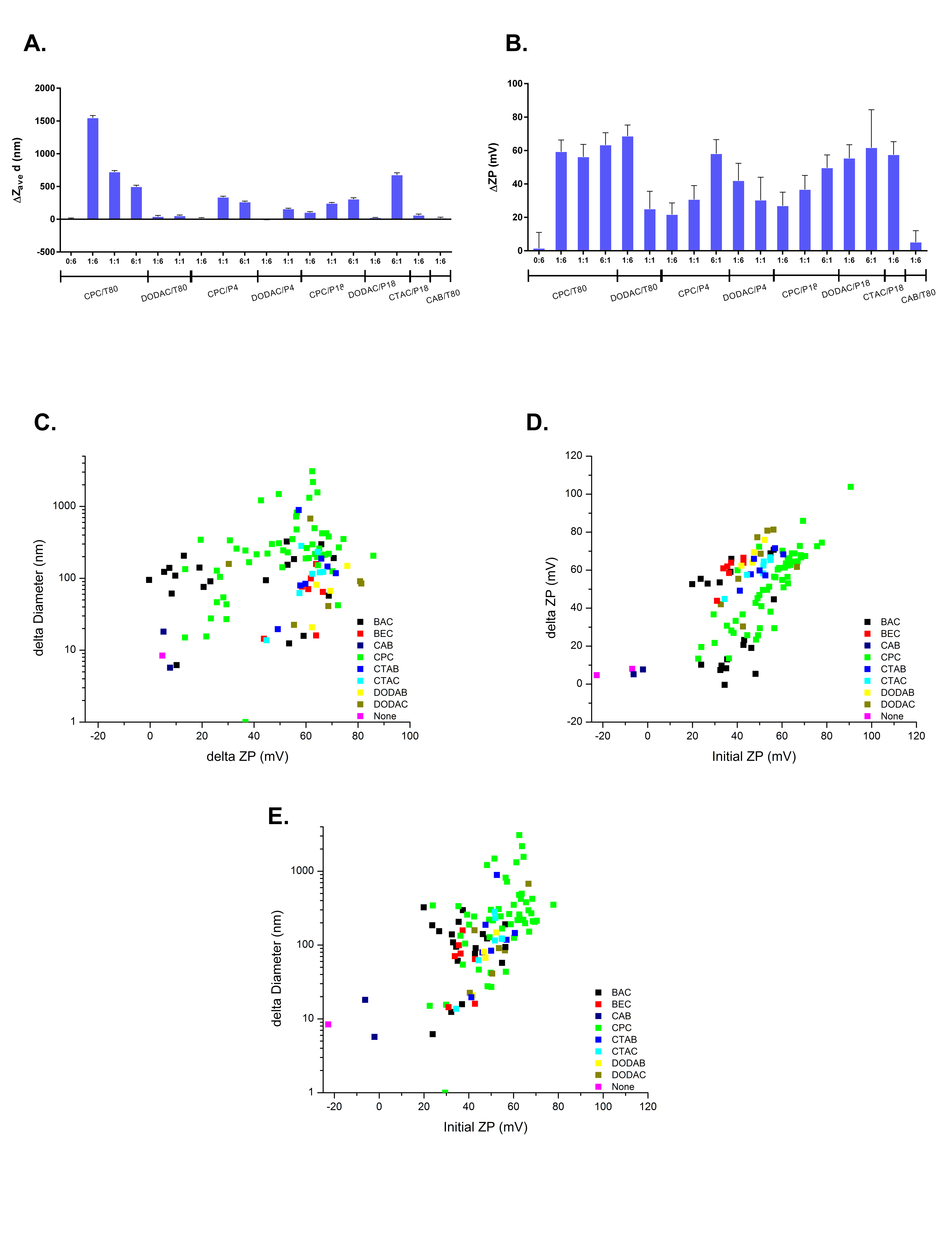

Supplement: S1 Fig — (A) Change in particle size and (B) ZP from Figs 1 and 2D scatter analysis of mucoadhesion properties for all NE formulations colored by cationic surfactant plotted by (C) ΔZave vs. ΔZP, (D) ΔZP vs. ZPinit, (E) ΔZave vs. ZPinit. (TIF) [file pone.0126120.s001.tif]

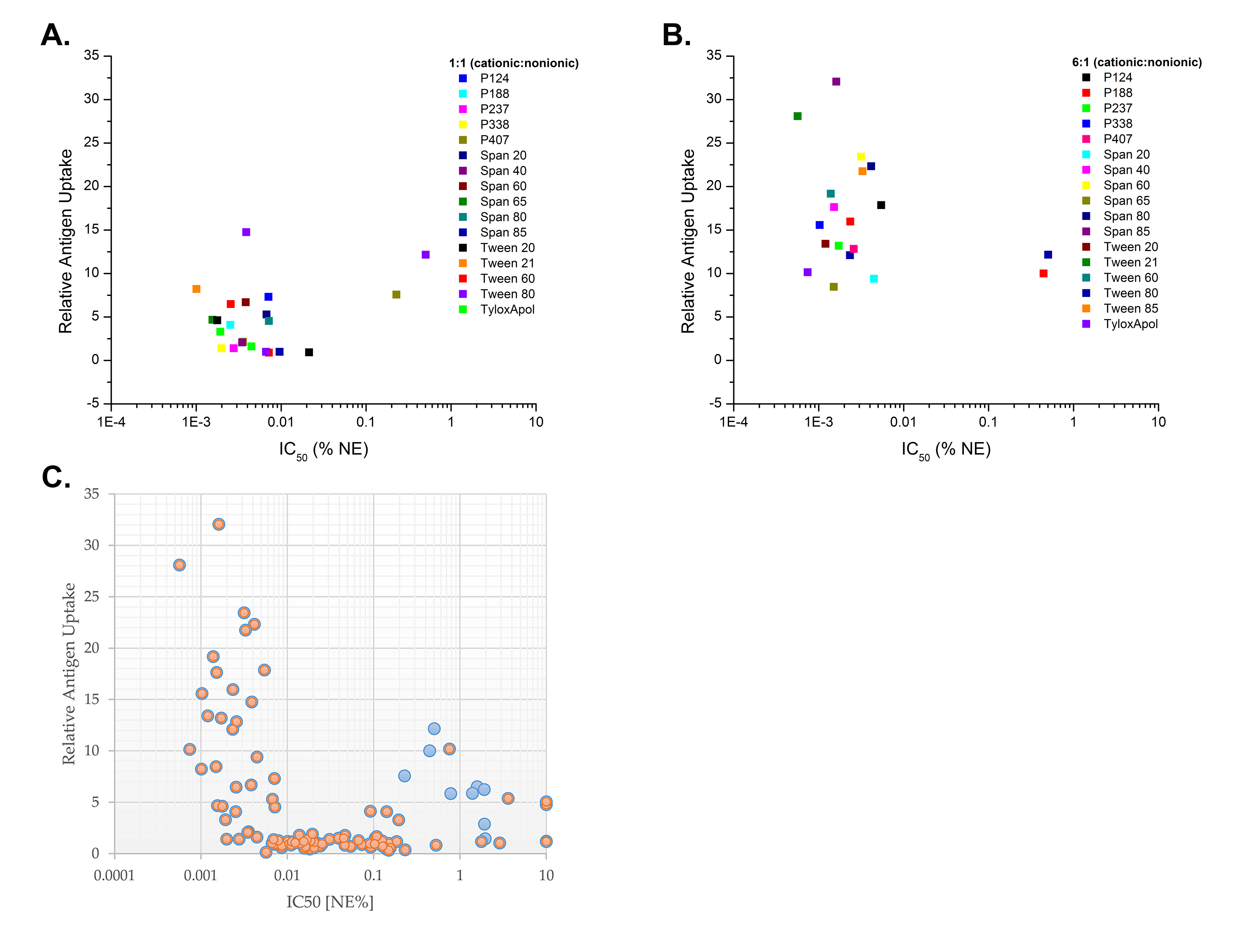

Supplement: S3 Fig — Scatter plots are shown for (A) 1:1 and (B) 6:1 NEs. (C) Scatter plots of antigen uptake vs. IC50 for all NEs tested at all ratios (same data as Fig 4A) colored by DODAC NEs (blue) and non-DODAC NEs (orange). (TIF) [file pone.0126120.s003.tif]

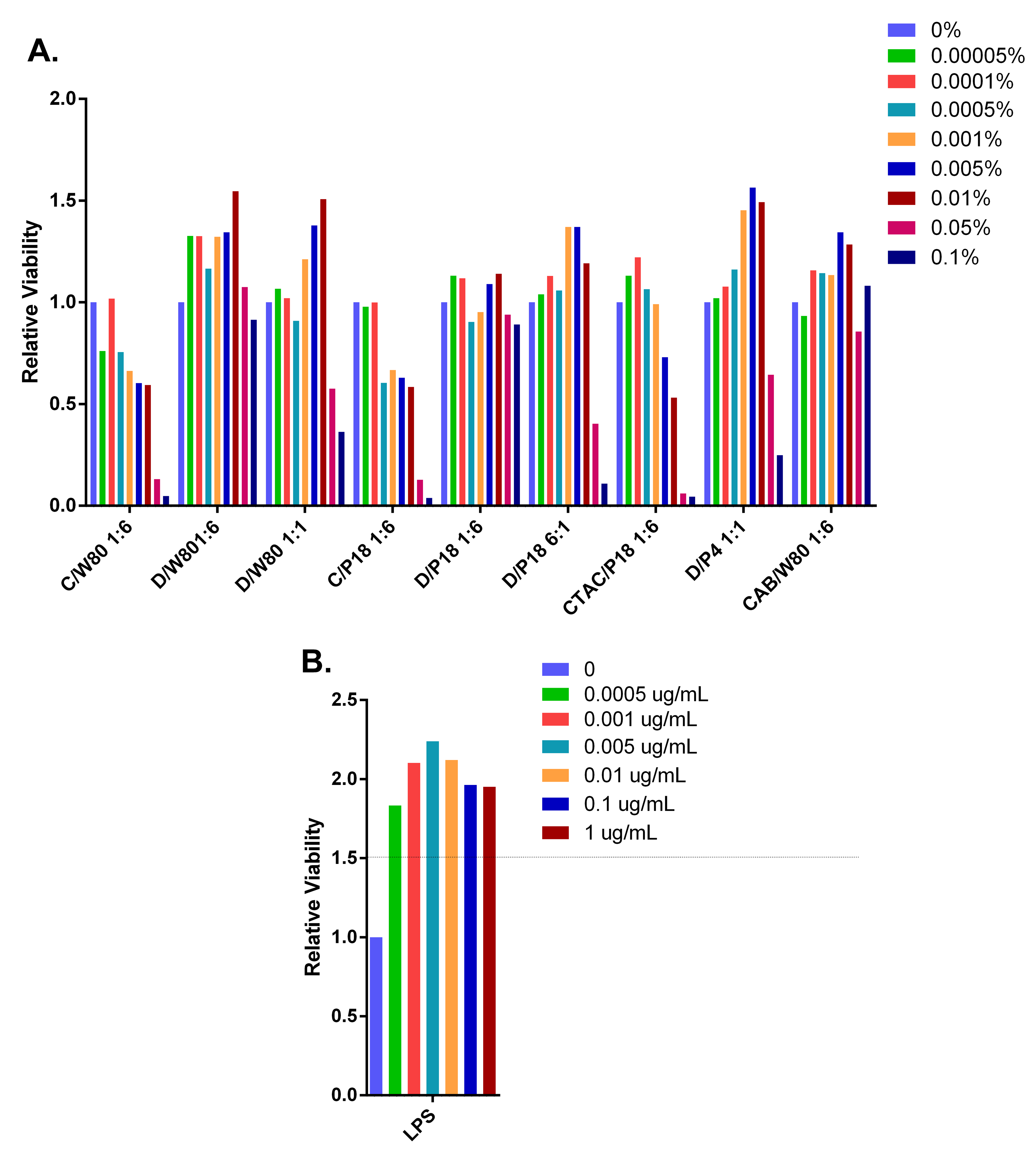

Supplement: S4 Fig — Relative viability of Raw-Blue cells used for NFκB activation studies as measured by an XTT assay after (A) 24h NE treatment or (B) LPS treatment from which supernatants were assessed for SEAP activity. Final treatment concentrations are expressed as % for NE and μg/mL for LPS. (TIF) [file pone.0126120.s004.tif]

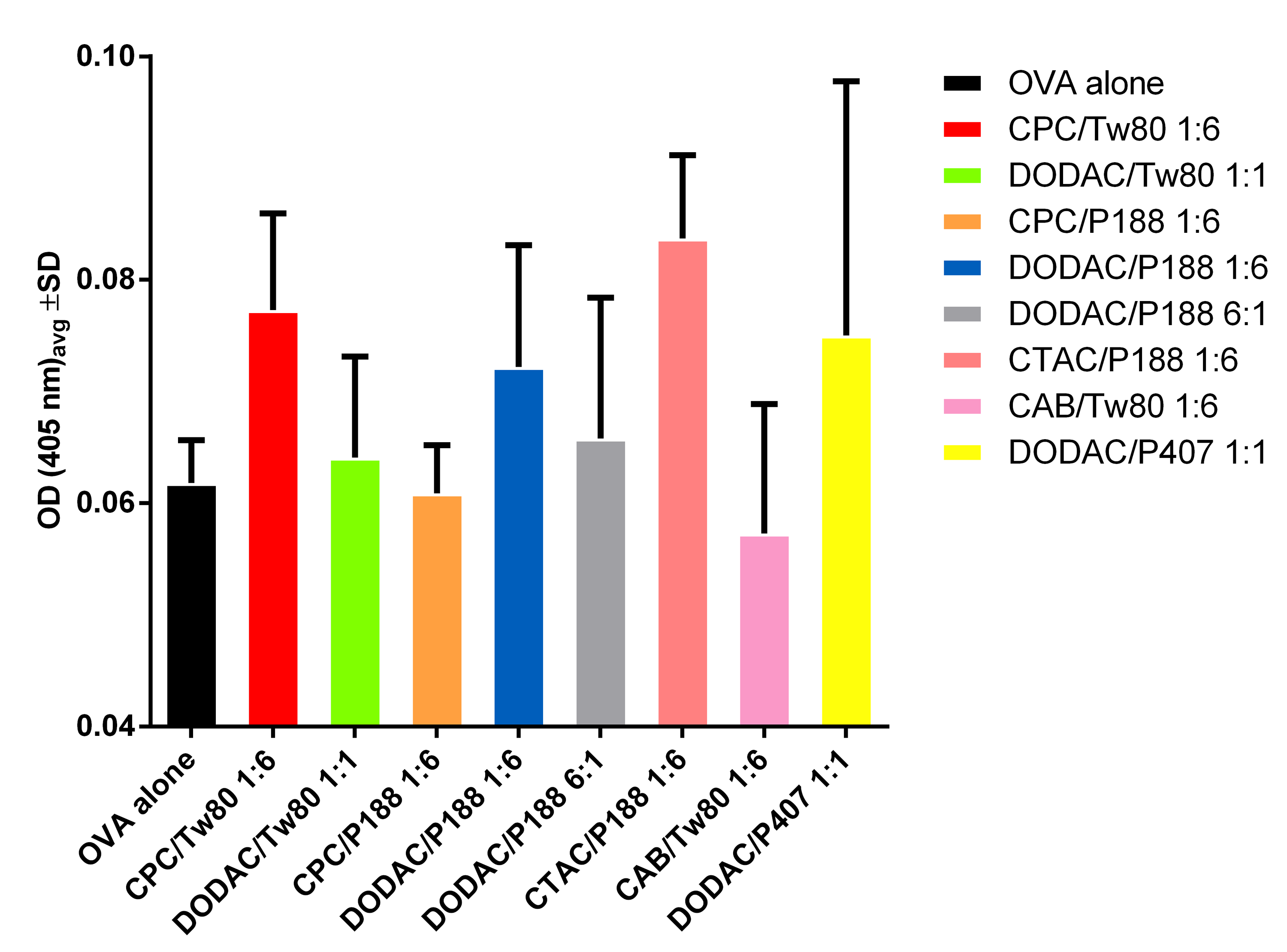

Supplement: S5 Fig — OVA-specific IgA from immunized mice measured in BAL is shown as OD at 405 nm ± standard deviation. (TIF) [file pone.0126120.s005.tif]

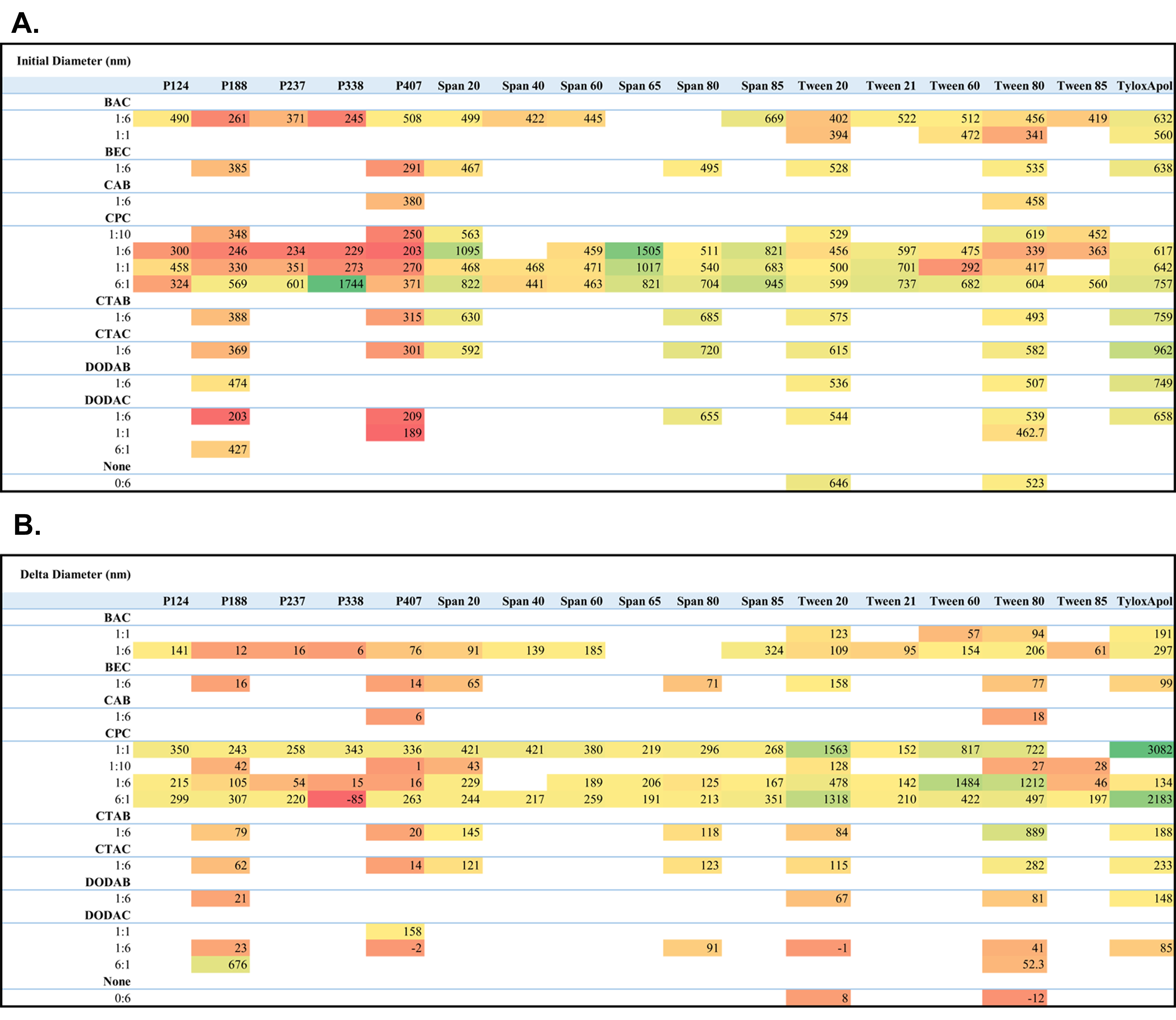

Supplement: S1 Table — (A) particle size (initial diameter) of NE alone, (B) ΔZave with mucin. (Zave, ΔZave, ZP, ΔZP, antigen uptake, cytotoxicity). (TIF) [file pone.0126120.s007.tif]

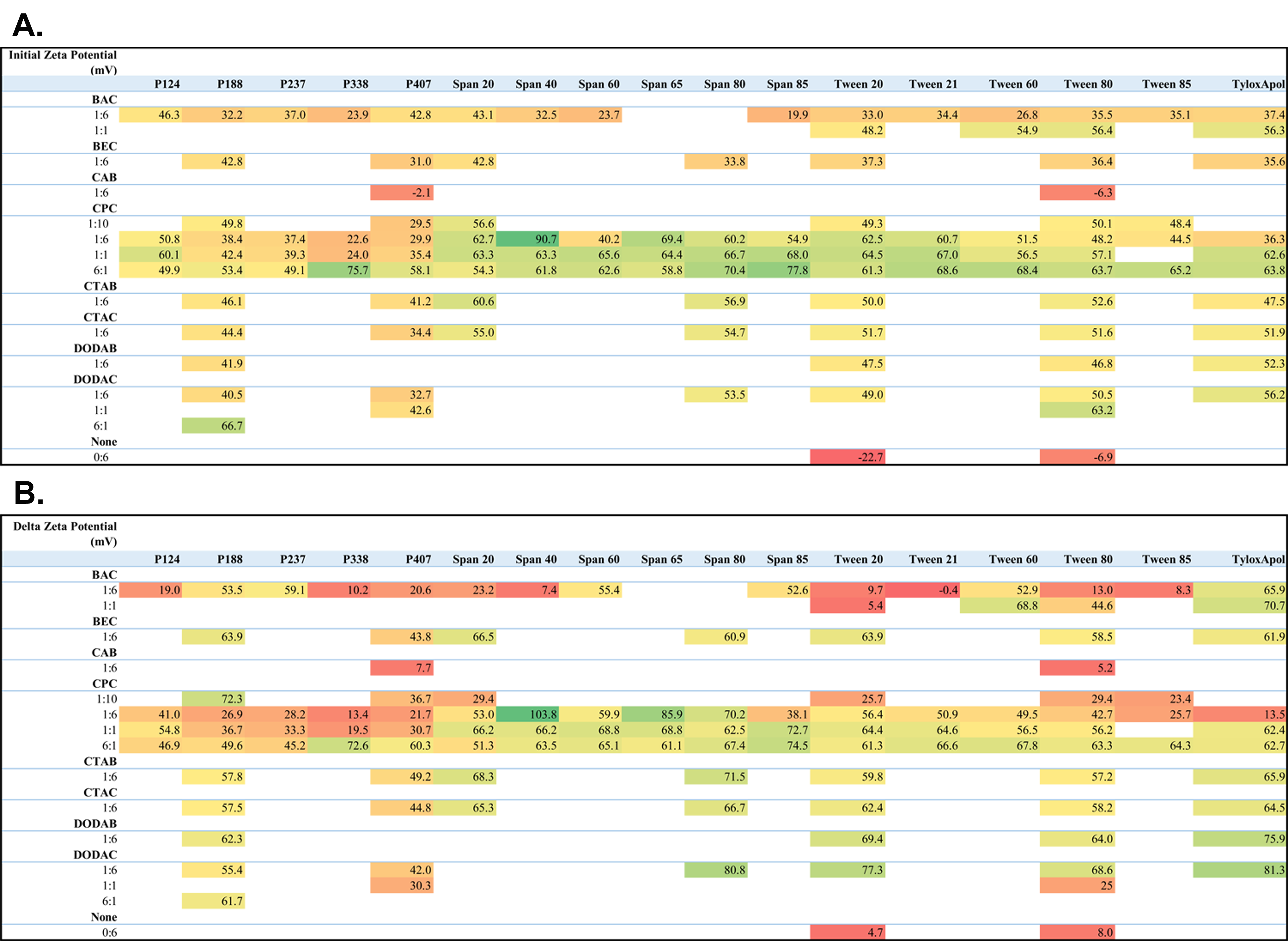

Supplement: S2 Table — (A) ZPinit, and (B) ΔZP with mucin. (TIF) [file pone.0126120.s008.tif]

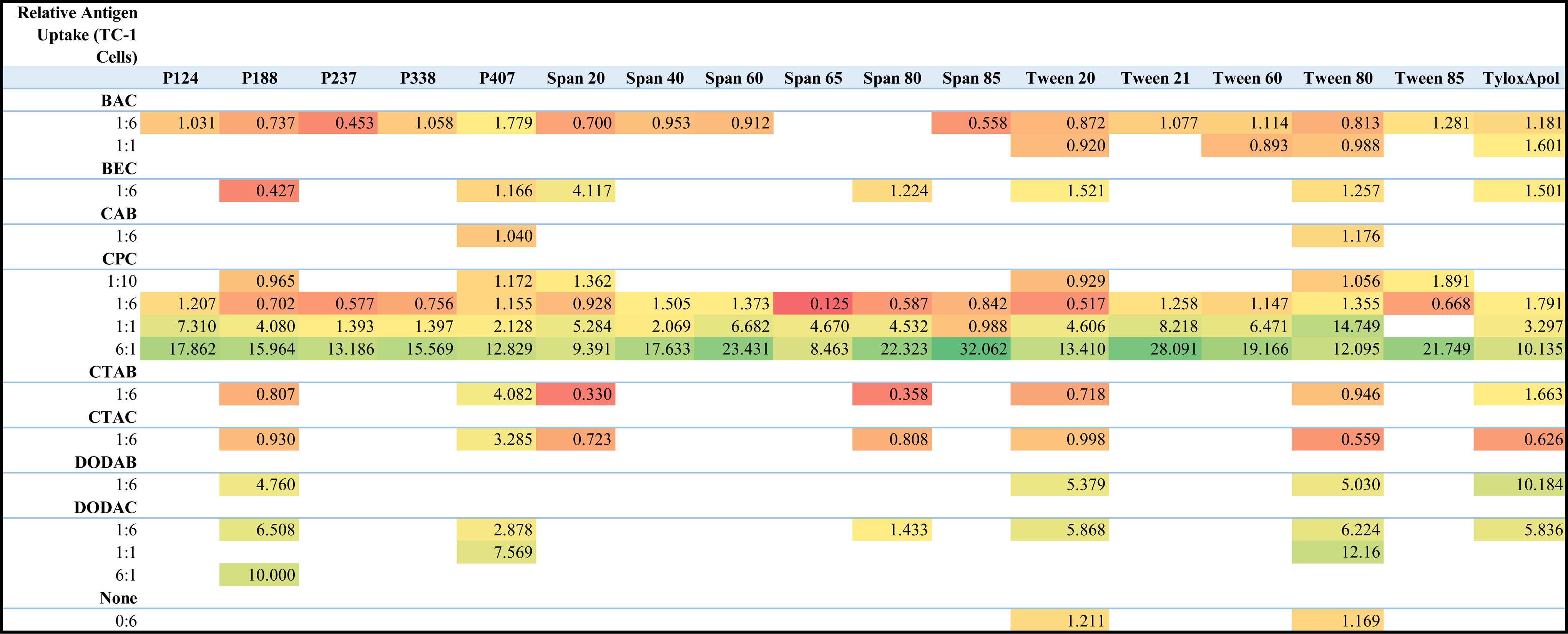

Supplement: S3 Table — relative antigen uptake in TC-1 cells. (TIF) [file pone.0126120.s009.tif]

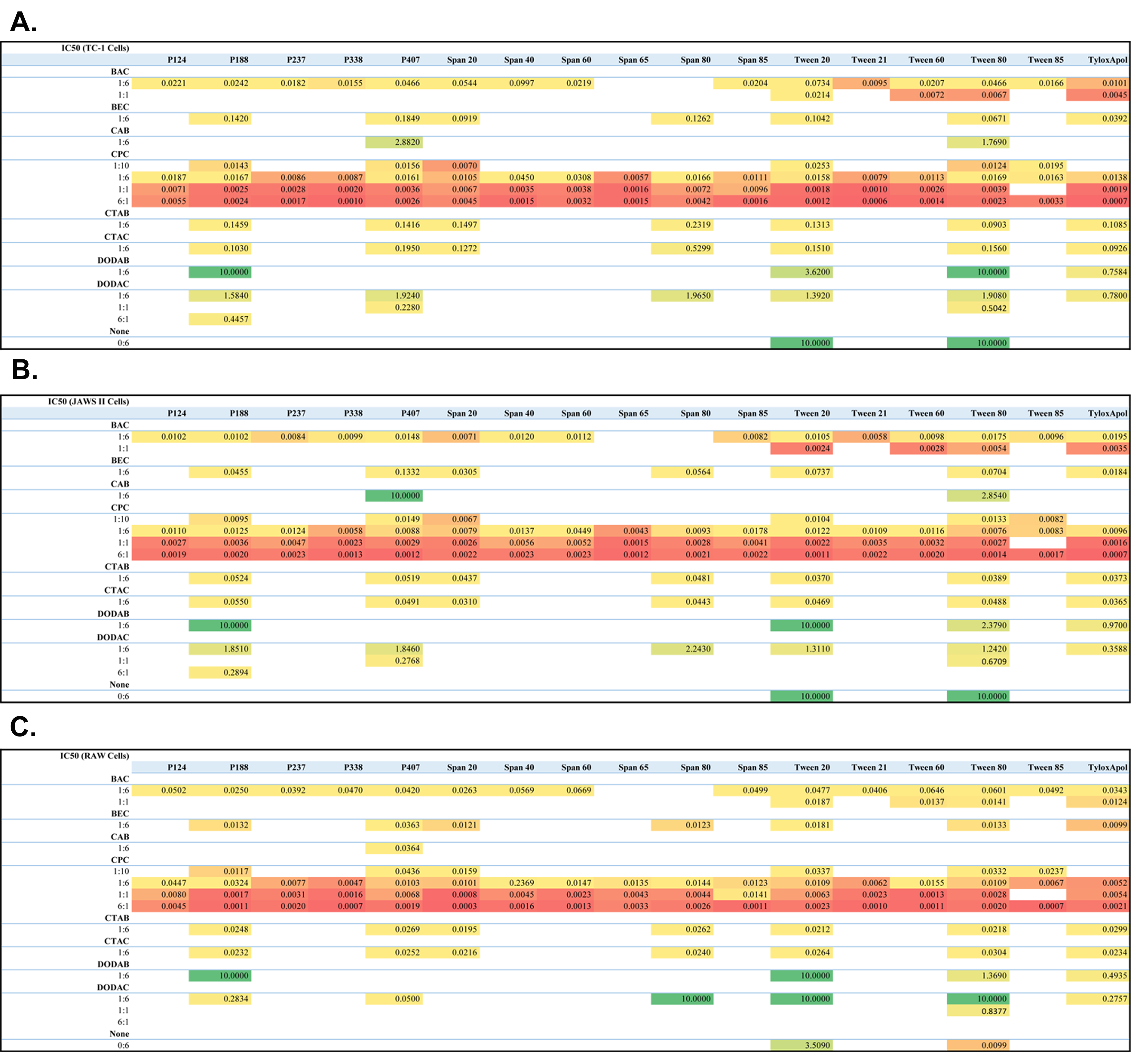

Supplement: S4 Table — cytotoxicity results expressed as IC50 values in (A) TC-1, (B) JAWS II, and (C) RAW cells. (TIF) [file pone.0126120.s010.tif]
